# Characterization of the Moroccan Barley Germplasm Preserved in the Polish Genebank as a First Step towards Selecting Forms with Increased Drought Tolerance

**DOI:** 10.3390/ijms242216350

**Published:** 2023-11-15

**Authors:** Maja Boczkowska, Marta Puchta-Jasińska, Paulina Bolc, Kinga Moskal, Szymon Puła, Adrian Motor, Katarzyna Bączek, Jolanta Groszyk, Wiesław Podyma

**Affiliations:** 1 Plant Breeding and Acclimatization Institute—National Research Institute, Radzików, 05-870 Błonie, Poland; m.puchta@ihar.edu.pl (M.P.-J.); p.bolc@ihar.edu.pl (P.B.); k.smolinska@ihar.edu.pl (K.M.); s.pula@ihar.edu.pl (S.P.); a.motor@ihar.edu.pl (A.M.); j.groszyk@ihar.edu.pl (J.G.); w.podyma@ihar.edu.pl (W.P.); 2 Department of Vegetable and Medicinal Plants, Institute of Horticultural Sciences, Warsaw University of Life Sciences SGGW, 159 Nowoursynowska Str., 02-776 Warsaw, Poland; katarzyna_baczek@sggw.edu.pl

**Keywords:** barley, drought tolerance, germplasm, DArTseq, SNP, diversity

## Abstract

In marginal, arid, and semi-arid areas of Morocco, crops are often exposed to multiple abiotic and biotic stresses that have a major impact on yield. Farmer-maintained Moroccan landraces have been shaped by the impact of very strong selection pressures, gradually adapting to the local ecosystem and obsolete low-input agricultural practices without improvement towards high yield and quality. Considering the increasing threat of drought in Poland, it is necessary to introduce germplasm with tolerance to water deficit into barley breeding programs. The aim of this research was a DArTseq-based genetic characterization of a collection of germplasm of Moroccan origin, conserved in the Polish genebank. The results showed that all conserved landraces have a high level of heterogeneity and their gene pool is different from the material developed by Polish breeders. Based on the analysis of eco-geographical data, locations with extremely different intensities of drought stress were selected. A total of 129 SNPs unique to accessions from these locations were identified. In the neighborhood of the clusters of unique SNPs on chromosomes 5H and 6H, genes that may be associated with plant response to drought stress were identified. The results obtained may provide a roadmap for further research to support Polish barley breeding for increased drought tolerance.

## 1. Introduction

At the end of the 1980s, British ecologist Norma Myers first presented the concepts of terrestrial biodiversity hotspots and identified the first 10 hotspots in the tropical forest area [1]. For a region to qualify as a hotspot, it must meet two criteria, i.e., it must contain at least 1500 endemic vascular plant species (>0.5% of the world’s total) and it must have less than 30% residual primary vegetation [2]. Currently, 36 regions worldwide are classified as biodiversity hotspots [3,4]. The Mediterranean basin is one of them. It has an area of 3,319,280 km^2^ and contains 1700 endemic plant species, making it the largest and third most important hotspot for plant diversity in the world [5]. Within the Mediterranean hotspot, the distribution of plant species richness is not uniform. A significant part of it is concentrated in the Anatolian, Balkan, and Iberian peninsulas and in north-west Africa [6].

Morocco is the richest and one of the most important reservoirs of plant diversity and speciation in the Mediterranean [7]. Some 3913 taxa belonging to 155 families, 981 genera, and 1298 subspecies have been identified, of which 878 taxa are endemic, i.e., about 22.5%. Of these, 540 species-level taxa belong to the dicotyledonous plant group and the remaining 59 to the monocotyledonous [8,9]. Underlying this richness is the country’s geographical location, its varied topography, geology, and climate. The fact that there is a dispersal route for plants and animals from and to the Macronesian Islands is also not insignificant. Moreover, Morocco is a land bridge between Africa and Europe. Numerous studies indicate the existence of strong floristic links between the ecoregions located on the two sides of the Strait of Gibraltar, i.e., in Andalusia and northern Morocco [10,11,12,13,14,15].

Morocco’s geographical location, which has almost 3500 km of coastline along the Mediterranean and Atlantic coasts and four mountain ranges in the High Atlas, the Antarctic Atlas, the Middle Atlas, and the Rif, results in the division of Morocco into at least four climate zones with significant variations in total rainfall and its seasonal distribution. Regions along the coast receive most precipitation from November to March, with amounts ranging from 300 to 700 mm. In the mountainous regions, winter precipitation can reach up to 2500 mm, and snow cover persists. In contrast, in the Saharan borderlands, annual rainfall does not exceed 200 mm, and the rainy season falls in autumn and spring [16]. Due to its geographical location, Morocco has faced droughts multiple times throughout history and this phenomenon appears to be a structural element of its environment [17,18].

In Morocco, traditional agricultural ecosystems belong to the agroforestry-pastoral type and are characterized by the cultivation of various traditional crops. Most farms, especially in the mountainous zones, are small and have a reduced usable agricultural area [19]. Traditional agriculture is still common, characterized by centuries-old farming practices that are sustainable and require low energy inputs. Rural landscapes combine environmental characteristics with traditional agricultural knowledge [20]. In marginal areas such as those found in Morocco, i.e., arid and semi-arid areas, crops are often exposed to multiple stresses that have a major impact on yields [21]. Under such conditions, landraces were shaped by the impact of very strong selection pressures of abiotic, biotic, and environmental factors, gradual adaptation to local ecosystems, and past agricultural practices [22]. Despite a noticeable shift towards the cultivation of modern cultivars since the early 20th century, landraces continue to be used by Moroccan farmers and can account for up to 95% of crops in semi-arid and marginal locations under the most adverse conditions [23,24]. The majority of cereal seed is sourced from an exchange network that includes farm maintained seed and seed obtained from local markets [25,26]. Farmer-maintained landraces are rarely subject to yield and quality improvement, and have the innate ability to produce sustained stable yields in agro-ecosystems characterized by high stress and low inputs [27,28].

Barley (*Hordeum vulgare* L.) is a cereal crop from the *Poaceae* family. It is one of the earliest domesticated species. Archaeological data indicate that this happened around 8000 BC in the Fertile Crescent region [29,30]. Although, a polyphyletic origin including independent domestication in the western Mediterranean region is possible [31]. Notably, the wild ancestor of *Hordeum spontaneum* was found in Morocco [32,33]. With the migration of people in the Neolithic, agriculture spread from western Asia south and west to Africa and north and west to Europe and further east to the Indus Valley [34]. Barley was the staple cereal in ancient times as confirmed by archaeological findings [35,36].

Barley is currently the fourth most economically important crop in the world. World barley production in 2021 according to official FAO data was 145.6 million tons. In Morocco, annual production has shown significant fluctuations even in the last decade. In 2021, a total of 2.7 million tons of barley was produced and it is the second most important crop after wheat [37]. Morocco has experienced drought in recent years which has an impact on cereal harvests [38].

Barley is used as feed, food, and in brewing [39]. Due to its nutritional value, its popularity as a staple food is increasing, especially in Africa and Asia [40]. It is a crop with a range of adaptation greater than other cereals. Its ecological range covers subtropical, temperate, and even arctic regions. It is cultivated successfully both at sea level and in high mountains, i.e., above 4500 m in the Andes and Himalayas, where wheat and oats cannot be grown [41,42]. Although its economic importance is considerably less compared to wheat, it replaces it in arid regions where precipitation is irregular, and rainfall amounts are insufficient to ensure satisfactory wheat yields. Numerous studies confirm barley’s high resistance to drought and salinity stress [42,43,44]. Barley is a typical crop of poor farmers in North Africa including Morocco, who grow it on mountain slopes, higher altitudes than other cereals, without using fertilizers or crop protection products. Under adverse environmental conditions, farmers use their landraces, which have undergone a process of natural selection over the centuries [45]. Numerous studies indicate that Moroccan landraces are a valuable source of resistance to abiotic and biotic stresses for modern breeding [44,45,46,47,48].

The collection of the National Centre for Plant Genetic Resources (NCPGR), i.e., the Polish Genebank, currently preserves a total of approximately 7330 accessions of the *Hordeum vulgare* species [49]. Among them, 185 are landrace/traditional cultivars. The most numerous group is made up of landraces from Poland, but as a result of two expeditions to Morocco carried out by the genebank team in 1986 and 1989, the second most numerous group is formed by Moroccan landraces. This collection, unlike the one with Polish origins, has not yet been characterized in terms of genetic diversity.

The aim of the study was to characterize the Polish collection of Moroccan origin accessions. Single nucleotide polymorphism markers (SNP) derived from DArTseq analysis were used to assess the diversity of the collection, as well as to determine the heterogeneity of the stored accessions and population structure. The results of the genetic analyses were cross referenced with eco-geographical data to investigate the influence of environmental parameters on genetic variation and to identify unique SNPs for accessions originating from locations with extreme values of eco-geographical parameters. By mapping to a reference genome, regions of allelic variation associated with extreme environmental conditions were identified and some genes within them were identified. This is the first step towards selecting pure lines that can be used in breeding programs for drought tolerance which is a rising problem in Poland.

## 2. Results

### 2.1. Eco-Geographical Data Summary

Eco-geographical data analysis was performed based on passport data containing information on the collection site of landraces. The landraces originated from seven regions of Morocco. In the north axis, the greatest distance of collection sites was 706 km and concerned PL 43350 and PL 42745, while in the east-west axis, the most distant accessions were PL 43350 and PL 43340, with 566 km between these sites. Landraces were collected at different altitudes above sea level. Accession PL 43340 was found at the lowest altitude, i.e., 100 m above sea level (a.s.l.) Three accessions came from locations above 1600 m a.s.l. (PL 42749, PL 42760, and PL 43349). The sites from which the landraces originated also differed in terms of 11 climatic parameters. The extreme values along with the accession number are given in Table 1. Annual and growing season data were included.

Analysis of variance ANOVA of both annual and growing season climatic conditions at the collecting sites showed no significant differences for the parameters, maximum temperature (tmax), climate water deficit (def), downward solar radiation flux at the surface (srad), and grass reference evapotranspiration (pet) (Table S1). Therefore, they were excluded from further analysis. A table with results of the analysis of the differences between the locations of the collection sites using Tukey’s (HSD) *post hoc* test can be found in Table S2.

Correlation analysis of eight eco-geographical parameters (Table S3) showed a very high (>0.9) positive correlation between precipitation (ppt) and water runoff (q) and actual evapotranspiration (aet). There was a high negative correlation between altitude and minimum temperature.

**Figure 1:**
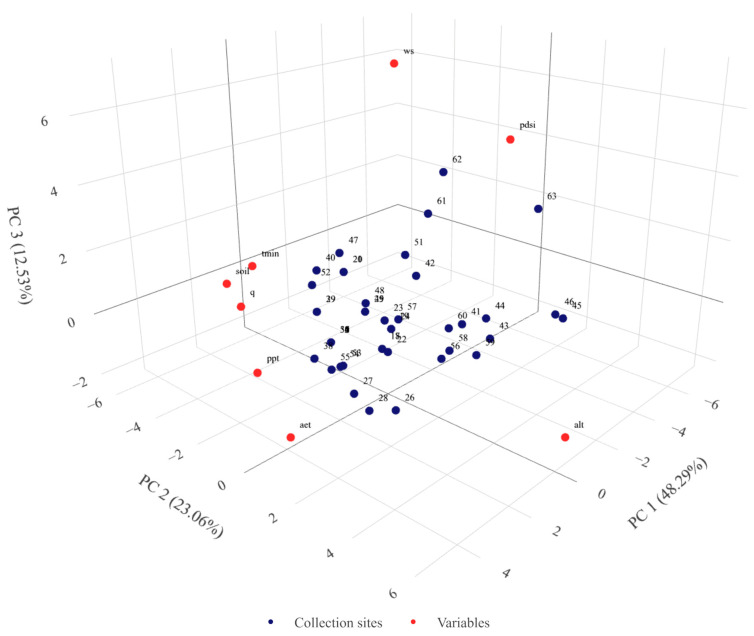
Graphical presentation of the principal component analysis results for eco-geographical parameters in the places of origin of the 47 Moroccan barley landraces. Results in the first three components’ system. Each point denotes one tested accession. Accession numbering according to Table S8. Rotatable 3D figure can be found in the Supplementary Materials (Figure S1).

PCA analysis performed based on eco-geographical parameters showed that the first three principal components explained 83.34% of the total variability. With the first principal component, four parameters showed a very high and high positive correlation, i.e., ppt (0.99), aet (0.95), q (0.95), and soil (9.0). With the second PC, tmin (0.93) and alt (0.93) were very highly correlated. The 3D plot based on the first three PCs (Figure 1) clearly shows six outlier points corresponding to the collection sites of PL 42766, PL 42767, PL 43340, PL 43350, PL 43351, and PL 43352 landraces. Four of them (PL 42766, PL 42767, PL 43351, and PL 43352) were characterized by maximum values of parameters such as q, soil, ppt, and aet. On the other hand, locations PL 43340 and PL 43350 were characterized by low values of the q parameter. In the case of PL 43340, the location had a maximum value of tmin and ws, and in the case of PL 43350, there was a maximum value of PDSI. The remaining sites form two major clusters comprising PL 42747 and PL 40982 collection sites and two minor ones comprised five and two locations.

### 2.2. Grain Morphometry

**Figure 2:**
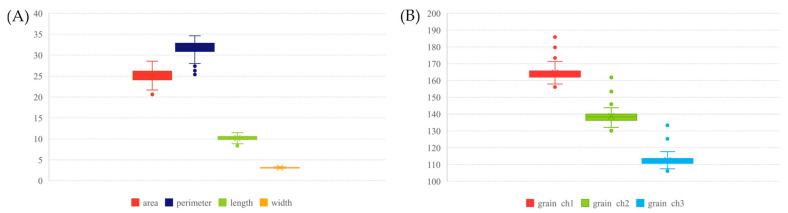
Grain morphology of the collection of 63 Moroccan barley accessions: (**A**) distribution of the parameter values describing grain size; (**B**) distribution of the parameter values describing grain color.

The study also considered the grain morphology of the studied objects. Parameters related to grain shape and color were compared. Variability among the seven parameters studied ranged from 3% to 8%. The variability within the breeding materials was higher than for the landraces. A summary for all parameters can be found in Table 2 and their distribution in the collection is shown Figure 2. Accession PL 42735 was characterized by the grain with the smallest perimeter and smallest length, but with the greatest width among the seed samples tested. The grain of accession PL 42380 had the smallest area and was the lightest, while that of PL 40414 was the darkest (Figure S2). Accession PL 42740 had the longest grain with the largest perimeter. Analysis of variance ANOVA showed that in terms of area, perimeter, and length, the grains of landraces and breeding materials differed significantly. For the other traits, the differences observed were not statistically significant.

Correlation analysis showed (Table S4) a very high positive correlation between area and perimeter and length and between these two parameters, as well. In addition, there was a very high and high correlation between all parameters describing seed color.

**Figure 3:**
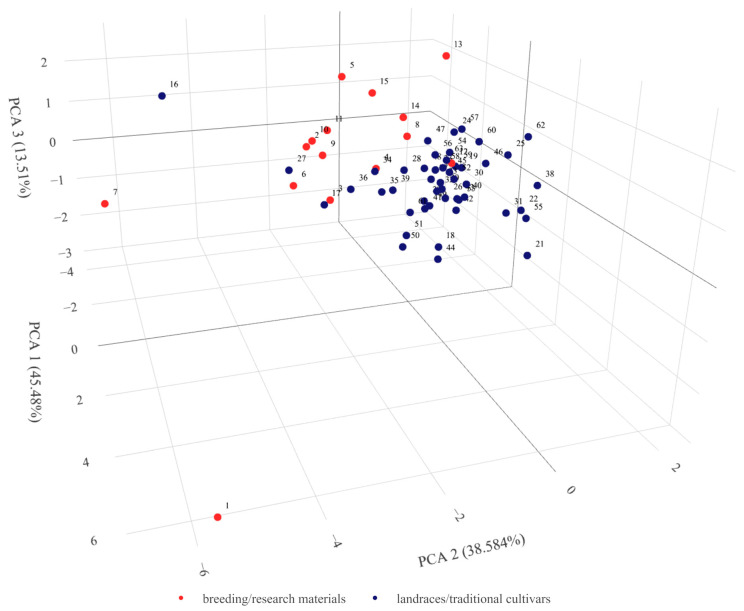
Graphical presentation of the principal component analysis results for morphometric parameters of the 63 Moroccan barley accessions. Results in the first three components’ system. Each point denotes one tested accession. Accessions numbering according to Table S8. Rotatable 3D figure can be found in the Supplementary Materials (Figure S3).

PCA analysis showed that the first three components explained as much as 97.57% of the total variability. All parameters were positively correlated with the first principal component. A strong correlation was found for area, grain ch1, and ch2. A perimeter was strongly positively correlated with the second principal component. Grain width and all parameters describing color were negatively correlated with PC2. While grain width was strongly positively correlated with the third principal component. In the 3D plot, it can apparently be seen that the groups related to biological status partially intermingle (Figure 3). Three accessions numbered 1, 7, and 16 in Figure 3, i.e., PL 40414, PL 42380, and PL 42735, respectively, were distant from the main point cloud. All three have been identified above as those with extreme values of the studied parameters.

### 2.3. Genetic Analysis

#### 2.3.1. Data Quality

**Figure 4:**
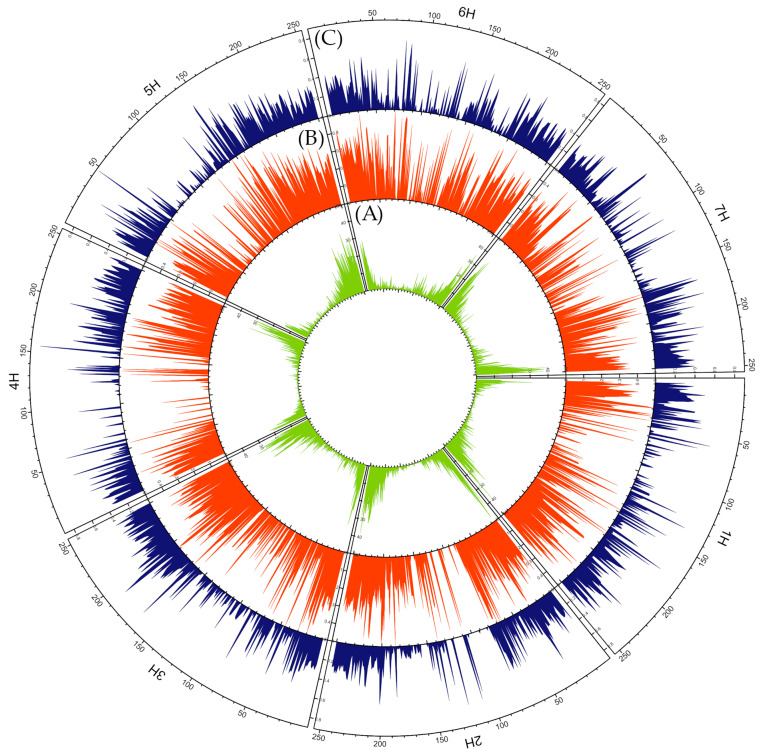
Circular overview of seven *H. vulgare* chromosomes based on DArTseq data acquired for 63 Moroccan barley accessions. (**A**) DArTseq loci distribution; (**B**) average polymorphism information content (PIC) distribution; (**C**) average observed heterozygosity (Ho) distribution. A sliding window approach with 500 kb windows, printed for 250 positions along the full length of barley chromosomes based on the genome assembly: IBSC_v2 [50] was applied.

Next-generation sequencing (NGS) provided reads for more than 77,000 loci. After filtering, 9737 loci for which the chromosomal location was known remained for further analysis (Table S5). On average, one analyzed locus corresponded to 0.47 Mbp of barley genome sequence. Across all chromosomes, a similar pattern of loci distribution was observed with a high frequency at the ends of the chromosomes and a reduction towards the centromere (Figure 4). The average PIC value for the loci analyzed was 0.26. More than 62% of the analyzed loci were highly polymorphic, i.e., had a PIC above 0.4 (Figure S4). The distribution of the PIC along the chromosomes was much more uniform. Nevertheless, on chromosomes 1H, 2H, and 4H, areas in the vicinity of the centromere were observed where the coefficient had a zero value. On 4H, this section was the longest (Figure 4)

#### 2.3.2. Mutation Types

About 60% of the identified mutations were transitions and 40% were transversions; therefore, the Ts/Tv ratio was 1.48 (Table 3). A>G and C>T transversions were the most common and together accounted for 52.4% of all mutations of this type. T>A and A>T transversions were the least frequent, accounting for 3% of transversions. The proportion of individual mutation types on chromosomes also showed variability. The highest proportion of transitions occurred on 5H (61.5%) and the lowest on 2H (57.5%).

#### 2.3.3. Diversity

**Figure 5:**
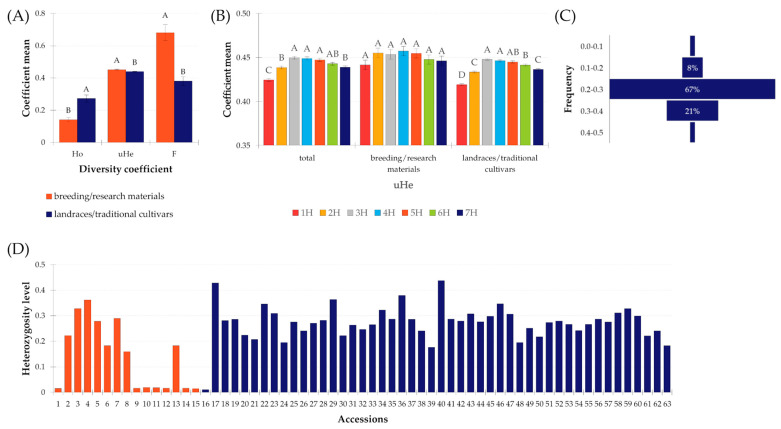
Summary of the diversity coefficient values for 63 accessions based on DArTseq data. (**A**) Observed heterozygosity (Ho), unbiased coefficient of variation (uHe), and fixation index (F) calculated for groups of accessions in accordance with their biological status; (**B**) unbiased coefficient of variation (uHe) on individual chromosomes according to biological status; (**C**) polymorphic information content (PIC); (**D**) heterogeneity level of 63 Moroccan barley accessions expressed by observed heterozygosity value based on SNPs derived from DArTseq analysis. Accessions numbering according Table S8.

The analysis of genetic differentiation was based on determining the values of three coefficients, i.e., observed heterozygosity (Ho), unbiased expected heterozygosity (uHe), and the fixation index (F). In the entire group of accessions studied, the aforementioned coefficients had values of 0.242, 0.443, and 0.455, respectively. Landraces had a significantly higher Ho coefficient value than the breeding materials. On the other hand, both uHe and F coefficient values were significantly higher in the breeding/research material group (Figure 5). The level of observed heterozygosity showed no significant differences on the seven barley chromosomes regardless of whether the entire set of accessions was analyzed or with division according to biological status. The F coefficient behaved similarly. On the other hand, significant differences were observed for the uHe coefficient. In the group of landraces, it reached the highest values at 3H, 4H, and 5H, and the lowest at 1H. This was also reflected in the parameters for the entire set of accessions. For breeding materials, the value of this parameter remained constant.

Diversity analysis of landraces according to the region of origin showed that the least heterogeneous accessions were those from the Tangier-Tétouan-Al Hoceïma region (0.144) and the most heterogeneous were those from the Fès-Meknès region. However, these differences were not statistically significant. In the case of uHe, all the regions studied had very similar values, ranging from 0.370 to 0.379, and of course the differences were not statistically significant. Among breeding materials, the highest Ho value was detected in accession PL 40979 (0.361) obtained at the genebank from the Plant Breeding Station Bąków. The lowest Ho value was found in PL 42694 obtained from the same source. Among the landraces/traditional cultivars, this coefficient ranged from 0.010 to 0.438 in PL 42735 and PL 42761.

#### 2.3.4. Population Structure

**Figure 6:**
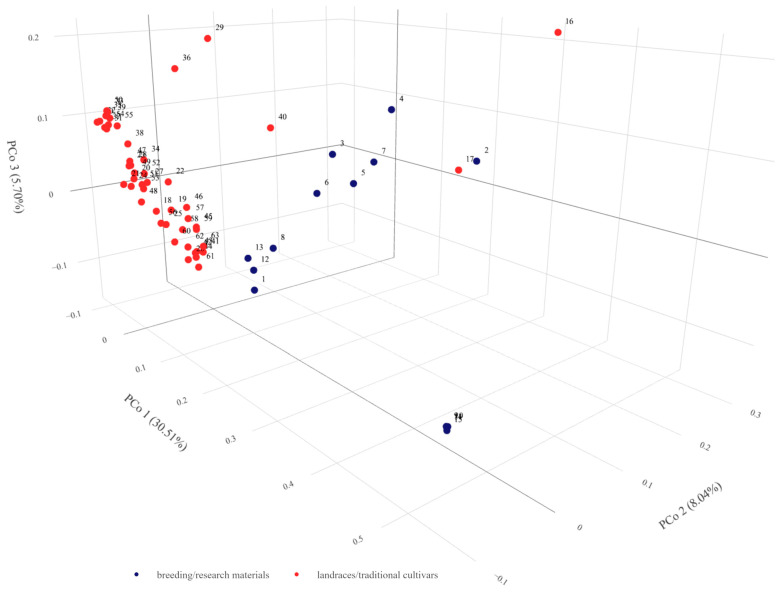
Graphical presentation of the Principal Coordinate Analysis results for DArTseq data of 63 barley Moroccan accessions with indication of their biological status. Results in the first three coordinates’ system. Each point denotes one tested accession. Numbering according to Table S8. Rotatable 3D figure can be found in the Supplementary Materials (Figure S5).

**Figure 7:**
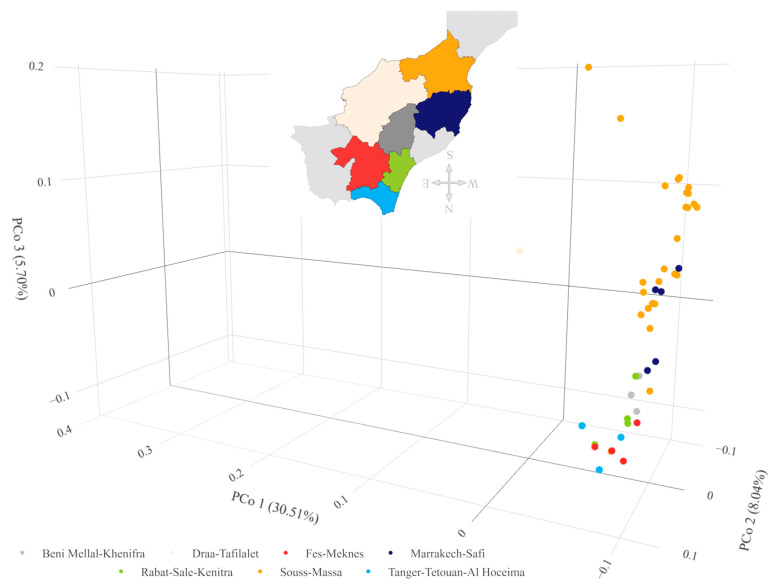
Graphical presentation of the principal coordinate analysis results for DArTseq data of 47 Moroccan barley landraces with indication of their region of origin. Results in the first three coordinates’ system. Each point denotes one tested accession. Numbering according to Table S8. Rotatable 3D figure can be found in the Supplementary Materials (Figure S6).

The average genetic distance in the set of accessions studied was 0.474 and ranged from 0.021 (PL 42379—PL 42380 and PL 42378—PL 42378—PL 42380) to 0.742 (PL 42735—PL 42751). Among breeding materials, the average genetic distance was 0.558 and among landraces 0.395, while among those with a known collection site location it was 0.384. Among accessions from the same location, i.e., collected in close proximity, the mean distance ranged from 0.309 to 0.699. In the landraces/traditional cultivars collection regions, the mean genetic distance was lowest for the Marrakech-Safi region (0.336) and highest for Rabat-Salé-Kénitra (0.642). The rest of the data can be found in Table S6. Analysis of molecular variance showed that 79% of the variation was found within groups related to biological status and 21% between them. PCoA analysis showed that the first three principal coordinates explain a total of 44.23% of the variability. In the 3D plot, the division between landraces and breeding materials can be clearly seen (Figure 6). It can also clearly be noted that the variation among the breeding materials was greater than within the landraces. Within the group of breeding materials, two subgroups of five and four objects were distinguishable. In addition, there were outlier accessions. The points corresponding to accessions 2 and 17 were located at almost the same place at a relatively large distance from the main groups. It should be noted that these accessions have two different biological statuses, i.e., one is a local form and the other is breeding material. A group of five breeding accessions (PL 42378, PL 42379, PL 42380, PL 42595, and PL 42694) were located at an even greater distance from the main groups. The points corresponding to them are very close to each other and actually overlap in Figure 7. Outlier accessions were also observed among the landraces. In addition to the previously mentioned, PL 42735 and PL 42736 were found at a significant distance from the main group. The remaining three accessions (PL 42749, PL 42756, and PL 42761) were located much closer to the main group. A chart considering only landraces in the context of the region from which they originated showed continuity in gene pool change between neighboring regions. It should be noted that gene pools in regions that are not directly adjacent to each other showed distinctness.

**Figure 8:**
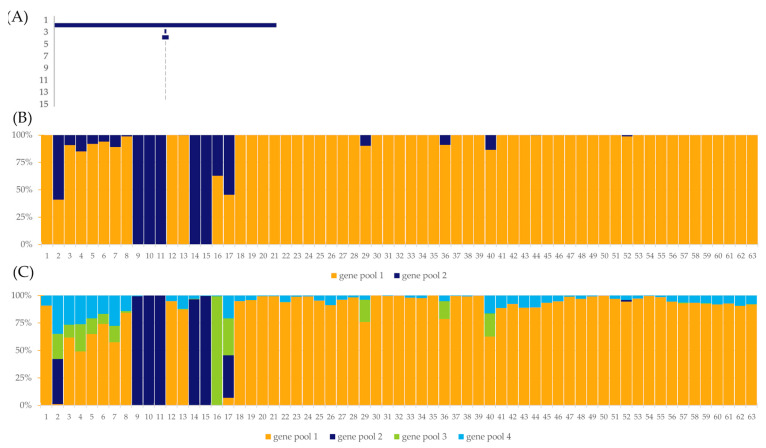
The results of 200,000 iterations of STRUCTURE software v 2. 3. 4 [51] for 63 Moroccan barley accessions based on DArTseq-derived SNPs data with K = 15 based on ad hoc measure ∆K [52,53], where K is the number of ad hoc clusters; each vertical bar represents one accession that is marked by order number according to Table S8. The length of the colored segment shows the estimated proportion of the membership of each gene pool in the cultivar genetic makeup. (**A**) The results of ad hoc measure of ∆K [52] generated by CLUMPAK software [53]; (**B**) primary genetic structure for 63 barley accessions at K = 2; (**C**) secondary genetic structure for 63 barley accessions at K = 4.

Analysis using Bayesian clustering showed that there was a distinct population structure in the surveyed set of accessions (Figure 8). According to the primary structure, the analyzed accessions were divided into two gene pools. The first pool mainly comprised landraces which, apart from accessions PL 42735 and PL 42736, were classified as pure at the 80% threshold. In addition, a part of the breeding materials (PL 40414—PL 40983, PL 42380, and PL 42593) belonged to the first pool. Five accessions defined as breeding materials were classified in the second gene pool. Three accessions were considered to be mixed. Further analysis of the population structure indicated the presence of a secondary structure containing four gene pools. In general, the distribution of accessions into major gene pools was similar. Almost all landraces represented a pure first gene pool, while five breeding materials the second pool. Accession PL 42735, on the other hand, was assigned to the third gene pool. And, accession PL 42736 considered three gene pools in almost equal proportions and a small admixture of the first pool similarly to PL 40672.

### 2.4. Association of Variability with Environment

The ANOVA showed essentially no significant differences in the values of the variation parameters in the landrace groups separated based on the eco-geographical data. Significant differences were only found for the uHe value at 5H for ws. The highest variation was in the group with the lowest mean ws during the growing season, and the lowest in the group where ws was between 3.16 and 3.62 m/s.

A comparison of the genetic and geographical distance matrices as well as absolute differences in eco-geographical parameter value matrices, using the Mantel test showed a negligible, i.e., <0.3, or non-significant correlation regardless of whether the genetic data were analyzed as a whole or by chromosome.

**Figure 9:**
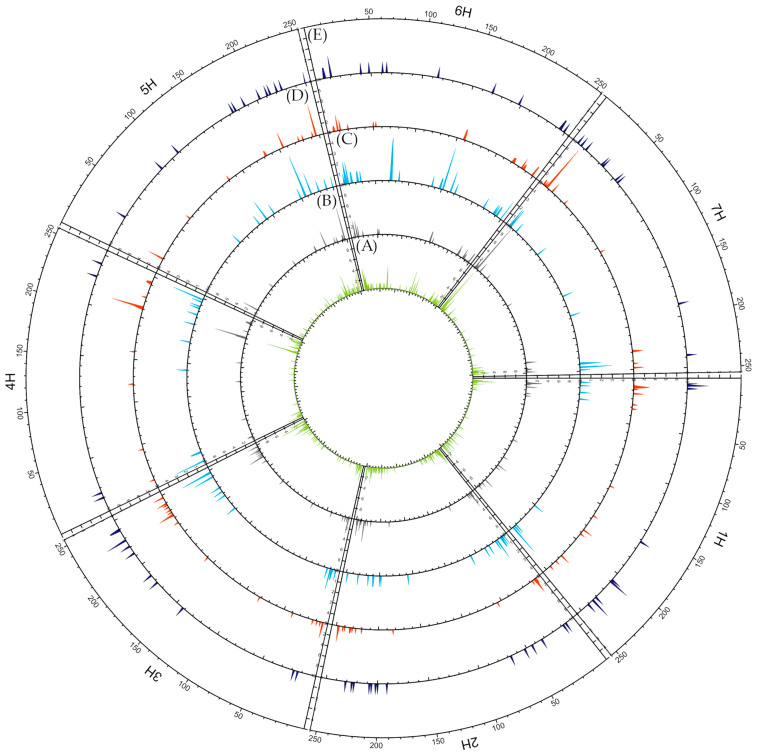
Circular overview of seven *H. vulgare* chromosomes. (**A**) Total number of unique SNPs of accessions collected at sites with extreme eco-geographical parameters value; (**B**) number of unique SNPs of accessions collected at sites with extreme soil moisture (soil) value; (**C**) number of unique SNPs of accessions collected at sites with extreme Palmer drought severity index (PDSI) value; (**D**) number of unique SNPs of accessions collected at sites with extreme precipitation (ppt); (**E**) number of unique SNPs of accessions collected at sites with extreme minimum temperature (tmin).

The investigated landraces were also subjected to analysis of loci uniqueness under extreme and significantly different environmental conditions (Table S2). A total of 592 unique alleles were identified to be associated with the impact of all studied eco-geographical parameters (Table S7). However, some alleles were found to be unique for more than one parameter. Therefore, the total number of unique alleles was lower and amounted to 396. The highest number of unique alleles (164) was detected for the soil parameter. The fewest unique alleles were associated with the altitude above sea level of the collection site, i.e., seven. The highest number of unique alleles was identified on chromosome 6H and the lowest on 4H. The pattern of distribution of unique SNPs was similar to that of all DArTseq loci, i.e., the frequency of unique SNPs was higher at the chromosome ends and decreases towards the centromere (Figure 9). A similar pattern of distribution of unique SNPs was observed for the ppt and soil parameters. On 4H, a significant number of SNPs unique to landraces collected from sites with extreme values of soil and ppt parameters was located at the end of the longer arm. SNPs unique to landraces derived from locations with extreme PDSI values were located on the longer arm of 5H. Unique SNPs were found at the end of the long arm. In addition, they also occurred in the pericentromeric region of 5HL. In contrast, on 6H, SNPs associated with PDSI were present at both ends, as well as in the pericentromeric region. Genes located in these regions can be found in (Table 4).

### 2.5. Combined and Comparative Evaluation

**Figure 10:**
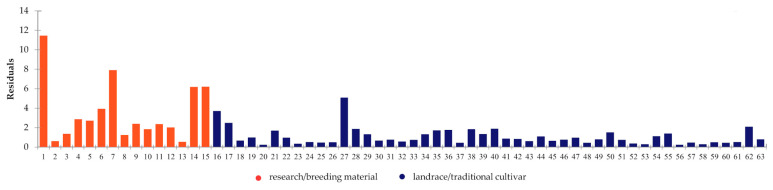
The result obtained during the Generalized Procrustes Analysis (GPA) showing the residuals of the accession after the transformations. Accessions numbering according to Table S8.

**Figure 11:**
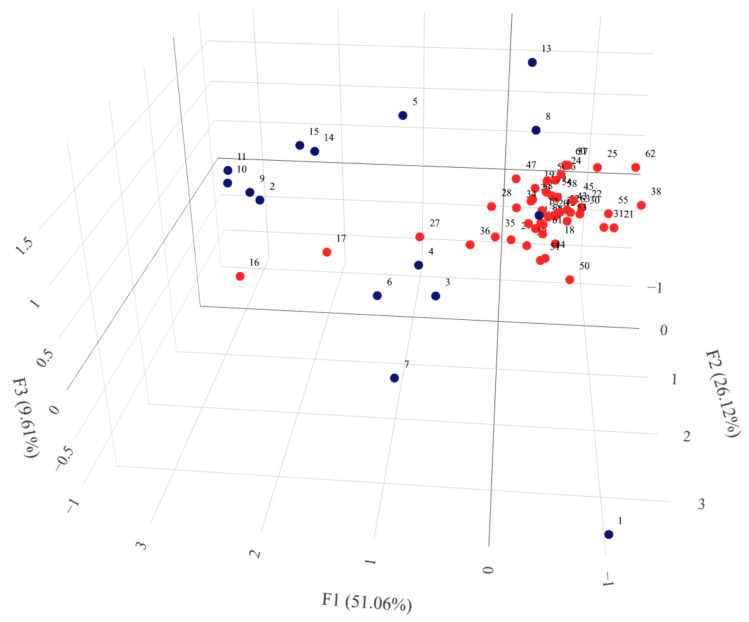
Graphical presentation of the Generalized Procrustes Analysis of genotypic and grain morphological data of 63 Moroccan barley accessions. Results in the first three coordinates’ system. Each point denotes one tested cultivar. Numbering according to Table S8. Rotatable 3D figure can be found in the Supplementary Materials (Figure S7).

Combining the results of the seed morphometric evaluation and SNP analysis was carried out using Generalized Procrustes Analysis (GPA). Based on the results of the Procrustes Analysis of Variance (PANOVA), it was determined that scaling and rotation significantly contributed to reducing the discrepancy between the results of the two analyses. Very similar residual values indicate a high consensus in the landraces group. In contrast, the level of consensus was much lower in the breeding material group (Figure 10). For both methods, the residuals were equal, but the value of the scaling factor indicates that the genetic data had a narrower scale range than the morphometric data. Based on the consensus test, the resulting configuration was found to be true and correct. Based on subsequent permutations, it was determined that the first three dimensions were sufficient to project the results and represent a total of 86.79% of the variation. In the 3D plot, the distinctiveness of the breeding materials from the landraces could be easily noticed (Figure 11). In addition, this group was characterized by greater variation, which was manifested by a wider scattering of points in space. Landraces form a relatively compact group from which only accessions PL 42747, PL 42736, PL 42735, PL 43351, and PL 42759 stand out.

## 3. Discussion

As a crop of major economic importance worldwide, barley is at the center of breeders’ attention. The new challenges posed to agriculture worldwide by global climate change, increasing pathogen pressure as a result of globalization, and the growing need to ensure food security, are leading to an intensification of breeding programs and the search for new sources of variability. There is ample evidence that intensive breeding programs conducted worldwide have led to genetic erosion of crop gene pools, resulting in a significant reduction in the diversity of cultivated genotypes [54,55,56,57,58,59,60,61].

Genebanks established for the collection, conservation, and use of dwindling biodiversity, including genetic variation in crop plants, are a source of novel, i.e., not used in modern breeding, genetic variants that confer increased tolerance to abiotic stresses and resistance to pathogens. The intensive development of high-throughput phenotyping and genotyping technologies over the last decade is providing opportunities to characterize germplasm on an unprecedented scale and with significantly improved resolution of results. This facilitates the introduction of desirable alleles from landraces and crop wild relatives (CWRs) into elite germplasm free of other genes that negatively affect their functional value. The global germplasm stock preserved in genebanks contains about 375,000 accessions, of which 34% are landraces and less than 3% are wild relatives [37].

Barley germplasm resources have been characterized for phenotypic and genotypic traits for many years at multiple sites worldwide, using a variety of techniques [62,63,64,65,66,67,68,69].

In the research presented here, a collection of accessions from Morocco, preserved in the Polish Genebank, was characterized. As described in the introduction, Morocco is located within a biodiversity hotspot, and diverse eco-geographical conditions and traditional agriculture have facilitated the emergence and survival of landraces adapted to multiple stresses [45]. High-throughput DArTseq analysis, which is commonly used to assess the genetic diversity of different plant species, was used here [70,71,72,73,74,75,76,77,78]. DArTseq analysis has previously been used to describe variation in other accessions from the NCPRG, i.e., native landraces and from several other countries, as well as cultivars bred and grown in Poland over the last 120 years [55,64]. To allow for comparison with the previous studies, the reads were mapped to the previous version of the barley genome reference assembly, despite its shortcomings.

### 3.1. Moroccan Accessions Diversity

The total variation of accessions of Moroccan origin was high (0.443). The total variation of the landraces was slightly lower (0.439). Compared to the previously analyzed accession groups, the differentiation of landraces was almost twice as high as that of Polish origin (0.226) and almost three times as high as that of Lithuanian origin (0.158). Moreover, the total differentiation of all previously studied landraces from several countries was significantly lower than that found in Moroccan landraces [64]. Also, the total variation during the whole period of barley breeding in Poland was lower compared to the accessions from Morocco. The range of the level of heterozygosity in individual landraces was very wide, i.e., from 0.01 to 0.438. Due to the specificity of the analysis, i.e., the use of pooled samples and the self-pollinating nature of the species, it can be assumed that the level of heterozygosity detected by the DArTseq method corresponds to the heterogeneity of the object. Apart from accession PL 42735, the remaining accessions had heterogeneity levels above 0.17, and as many as 23% above 0.3. Compared to previously studied landraces from the Polish Genebank, these accessions are definitely more heterogeneous [64,79]. This may be related to the initial higher variability of genotypes within individual accessions. Due to the stressful environmental conditions and the traditional low-input cultivation method, higher heterogeneity increases the uniformity and predictability of yield [80,81,82]. Also, important for the heterogeneity of the samples stored in the genebank is whether a sufficiently large, representative sample of mature seeds could be collected from the landrace site [82,83,84]. Another critical factor is the accuracy of the reproduction of the seed sample in the genebank. This is influenced equally by the human factor, i.e., the correct size of the seed sample sown, appropriate agro-technology and proper harvesting, post-harvest handling and storage, and the environmental factor during reproduction. Varying climatic conditions, pressure from pathogens, agrophages, and wildlife can contribute to the loss of initial variation in landraces [83,84]. All of these factors contribute to genetic drift, which is defined as random variation in allele frequencies between generations within populations due to sampling error [85,86]. Genetic drift results in a loss of genetic variation within and between populations and increased homozygosity of individuals [86]. Therefore, based on the results obtained, it can be concluded that the phenomenon of genetic drift had little or no effect on the Moroccan landraces collected during the Polish Genebank expedition. The significantly lower heterogeneity of accessions from Poland and Lithuania is probably related to the less demanding environmental conditions found in both countries compared to Morocco. During the collection period of landraces in Poland and Lithuania, drought stress occurred at low intensity at their collection sites [87,88]. However, in recent years, drought stress has been observed with increasing frequency and severity, especially in the spring period [89]. Forecasts predict an intensification of this phenomenon in Poland in the future [90].

A comparison of the level of genetic variability of landraces between different regions in Morocco showed no significant differences. This result could be due to the occurrence of different landraces in different regions with overlapping gene pools, or to the occurrence of the same alleles but at different frequencies. However, a more likely reason was the use of grouping based on the administrative division of the country rather than geographical regions or climatic zones. This division was not possible due to the lack of exact locations of the collection sites. Geographical coordinates are available in the database with minute accuracy; therefore, the location of the collection site was determined with an accuracy of up to 1 km. In addition, accurate climate maps and meteorological data for the collection period were not available. The analyses used monthly averages from 1958 to 2020 approximated with a spatial resolution of about 4 km. However, the result of the PCoA analysis clearly indicated differences at the genetic level related to the region of origin of the landraces. There was a clear distinction between landraces from the north-facing, coldest Mediterranean coastal region of Tanger-Tétouan-Al Hoceïma and those collected further south in one of the hottest regions of Morocco, Souss-Massa, located in the Sahara foreland.

### 3.2. Genetic Structure

The results indicated that there was a strong genetic structure in the accessions studied. Two gene pools were clearly distinguished. The first was characteristic of the landrace and the second of the five breeding materials. Due to the dominance of the first gene pool in as many as nine accessions with breeding material status, it can be assumed that they originated from landraces from Morocco. The degree of internal variation varied widely from 0.015 to 0.362, indicating that these materials had undergone different degrees of selection. Accessions PL 40414 and PL 42593 had a level of heterogeneity indicating that a single genotype was present in the material studied. Analysis of the passport data showed that PL 40414 was named Rabat and came to Plant Breeding Station Strzelce (POL054), from the National Plant Germplasm System (NPGS) US Genebank (no. CIho4979). According to passport data, the biological status of this accession is uncertain, and it has been in the collection since 1927. It is not possible to determine whether the homogeneity of this accession is due to a deliberate effort by the breeders at the Institut National de la Recherche Agronomique (INRA) Morocco or whether the differentiation was lost at a later date, since this accession was acquired at the NCPGR in 1972. The origin of PL 42593 is unknown. The Plant Breeding Station Bąków (POL020) did not provide information to the database on how the seeds were obtained.

Accession PL 40672 showed the presence of both gene pools in almost equal proportions. Interestingly, a very similar structure was also identified in PL 42736. PL 40672 was acquired by the NCPGR from Leibniz Institute of Plant Genetics and Crop Plant Research (DEU146) and appears in the local database under accession number HOR 3877. According to the passport data, it is an advanced cultivar bred at INRA in Morocco. Analysis of the passport data in several genebanks shows that this cultivar was definitely bred before 1964. It was acquired by the Polish Genebank in 1973, while it has been in the collections of the IPK Genebank, the USDA Plant Industry Station in Beltsville, and the John Innes Center since 1964. Interestingly, the similarity between the two accessions was also shown by PCoA analysis, i.e., the corresponding points were very close to each other. It should be noted that although these accessions show great genetic similarity, they differ at the level of seed morphology. PL 42736 was collected during the NCPGR expedition to Morocco. Since the collectors relied heavily on information provided by farmers on the farms where the seed was collected, there is a possibility of misinformation. When old cultivars have been grown on farms for several decades, information about their origin and name is lost in people’s memory. Similar examples were found for landraces of oats in Poland and landraces of barley in Lithuania [64,91].

An interesting result was also obtained for accession PL 40777, which was transferred to the NCPGR from POL020 in 1972. According to the EGISET database, it is a breeding material with the name Rabat 071. The database also indicates that the accession may have been obtained from the NPGS, as the accession number CI 9776 also appears in the passport data. However, based on DArTseq analysis, this accession is characterized by a high level of heterogeneity, while passport data from the NPGS indicate that it is currently a pure line. However, it is not known when the pure line was derived from the originally acquired landrace. Unfortunately, it is not possible to verify the degree of similarity between the two accessions without comparison with the original sample obtained from the NPGS. However, it can be assumed that the accession stored at NCPGR was derived from crosses with other genotypes in the breeding program conducted by POL020. This is indicated by its considerable similarity to some of the accessions from this donor. This similarity was evident in both the PCoA and population structure plots, especially when analyzing the secondary structure.

Doubts about its origin were raised by accession PL 42735. The EGISET database shows that it originated in Morocco. However, further information from the expedition to Morocco is missing. Based on DArTseq analysis, it is clearly genetically divergent from Moroccan landraces, as shown in the PCoA plot, and represents a gene pool separate from Moroccan landraces. The name of accession E 1012 in the passport data, i.e., sample number of the expedition, indicates that it may have originated from the 1984 expedition to eastern Poland, since according to the original notebook of the 1984 expedition to Morocco, the expedition numbers start with E 1025. Furthermore, this accession is characterized by a very high homogeneity, which may indicate that it is either a breeding material or that the accession was isolated during the expedition from another collected sample in which it was an admixture. Unfortunately, the original notes from the Polish expedition have not been preserved in the NCPGR archives. Therefore, it seems necessary in the future to compare the genetic data of this accession with material representing the Polish gene pool from the Lublin area and cultivars grown at that time.

Based on the genetic data, it could be assumed that at least three independent crossbreeding programs were carried out in POL020 using the Moroccan gene pool. The first program is represented by accessions PL 40414, PL 40983, PL 42593, and PL 42594. The probable Moroccan donor could have been the cultivars ‘Rabat’ and/or ‘Marzaga’. The second program is represented by accessions PL 40777—PL 40982. Here, it can be assumed that the probable Moroccan donor was the original pure line, or the landrace Rabat 071 obtained from the USA. The third program is represented by accessions PL 42378—PL 42380, PL 42595, PL 42694. Unfortunately, from the passport and genetic data, it is not possible to determine which Moroccan accession was used to cross and select the most likely breeding lines acquired by the Polish Genebank, since all these accessions are characterized by a very high homogeneity. The donor of the material to the genebank did not provide any information on their pedigrees.

### 3.3. Water Deficit Adaptation and Its Potential for Breeding

Barley shows considerable adaptation to soil stresses such as drought and salinity, which limit growth and yield [92]. Farmer-maintained landraces have undergone little selection and improvement for yield and grain quality, and their variation is the result of adaptation to local biotic and abiotic stresses [22,27]. Studies have shown that the yield of landraces under unfavorable conditions can be comparable to or better than that of improved cultivars [45,93,94,95,96]. As discussed in the introduction, Morocco is a country with a long history of drought; therefore, it is reasonable to assume that landraces developed under these stressful conditions need to be adapted to grow and yield under water deficit conditions. Studies have shown that landraces from regions with low or even extremely low annual rainfall can be valuable genetic resources for improving drought tolerance in barley [97,98,99,100,101]. Plant responses to water deficit involve complex mechanisms ranging from gene expression to ecosystem processes [102]. Plants can alter physiological and biochemical traits in response to prolonged drought, with mutations and gene modifications underpinning this [103]. Multiomic comparative analyses identified 26 genes associated with water deficit resistance in the XZ5 genotype of wild Tibetan barley (*Hordeum vulgare* L. ssp. *agriocrithon*) [104].

Due to the fact that the research presented in this article was conducted on a small number of accessions, most of which were heterogeneous, and the lack of phenotypic observations, it was not possible to perform association mapping for traits related to drought tolerance. By comparing accessions from locations with extremely different PDSI coefficient values, unique alleles were selected. The PDSI coefficient is commonly used to monitor the extent and severity of drought [105]. It is calculated using data on precipitation and evapotranspiration over time, water runoff and moisture supply, and soil water-holding capacity at the study site [106,107]. A total of 129 unique alleles associated with PDSI were identified. The largest number was located on chromosome 6H. On both 5H and 6H, clusters of unique SNPs were located in pericentromeric regions, which are characterized by low diversity and low frequency of SNPs detected by DArTseq. On these two chromosomes, an attempt was made to identify genes located in the vicinity of the SNP cluster. The fragment sequences obtained after DArTseq analysis were mapped to the IBSC_v2 reference genome [50] for future co-analysis and comparison with previously obtained results for landraces from the KCRZG collection.

A total of 11 genes located near groups of SNPs of interest, at least some of which may be related to the plant’s response to drought stress, were identified based on the annotation of the reference genome.

A gene encoding a Trichome birefringence-like N-terminal domain-containing protein has been located on chromosome 5H. Genes encoding these proteins are associated in plants with tolerance to abiotic stresses and defense against pathogens [108]. In *Arabidopsis*, their role has been well documented in the initiation of trichomes [109]. The density of trichomes, together with leaf waxiness in barley, may reduce water loss and protect against drought for prolonged periods [110,111].

A gene encoding a putative esterase/lipase/thioesterase family protein is also identified on the same chromosome. The acyltransferase esterase/lipase/thioesterase family from *Arabidopsis thaliana* L. are involved in the deposition of free phytol and free fatty acids as phytyl esters in chloroplasts. This process is associated with the maintenance of photosynthetic membrane integrity during drought stress and ageing [112]. In *Brassica juncea* L., differential hypomethylation of esterase/lipase/thioesterase family gene promotors in response to drought was detected. The overall hypomethylation of promoters of genes of this family may counteract drought stress by maintaining an active photosynthetic machinery [113].

On chromosome 6H, the gene encoding Dehydrin 12 (*DHN12*) was located in a region of unique SNPs. Dehydrins are expressed in cells during periods of low water content and are able to improve desiccation tolerance by protecting membranes, proteins, and DNA [114]. However, *DHN12* is the only gene in this family that is not expressed in vegetative tissues under dehydration, salt, or cold treatment. *DHN12* is expressed only in developing grains [115].

Also, located on 6H is the gene encoding cinnamyl alcohol dehydrogenase (CAD), a key enzyme in lignin biosynthesis [116]. Studies have shown that an increase in CAD activity leads to an increase in cinnamyl alcohol synthesis, and therefore is a specific marker of lignification. Increased lignification improves the mechanical strength of root cells and helps alleviate osmotic stress [117,118].

The genes encoding histone H2B and monogalactosyldiacylglycerol synthase were also located in the region of unique SNPs on 6H associated with extreme PSDI values. In eukaryotic cells, H2B is one of the four major histone proteins involved in chromatin nucleosome structure [119]. In wheat, a drought stress-responsive gene of the H2B family was identified. Its expression was significantly increased under drought stress and knockout mutants were significantly more sensitive to water deficit [120]. The upregulation of H2B under drought stress during grain filling in a water deficit tolerant barley cultivar from Egypt indicated that histone assembly and disassembly are one of the important factors regulating the transcription of some genes [121]. Monogalactosyldiacylglycerol (MGDG) is one of the two major galactolipids present in the photosynthetic membrane of many algae and higher plants [122]. It is one of the major components of the thylakoid membrane and is essential for chloroplast biogenesis and a photoautotrophic growth [123]. MGDG synthase is overexpressed in rice in association with waterlogging, drought, and salinity stress [124]. In maize leaves, MGDG synthase was upregulated under drought conditions, and changes in galactolipid composition have been implicated in alleviating leaf senescence in response to water deficit [125].

It can therefore be assumed that the diversity in the above regions is related to adaptation to drought stress. However, the results obtained can only provide a roadmap for further research. The drought stress tolerance of landraces from Morocco coupled with yield potential should be evaluated and compared under controlled conditions. The level of heterogeneity of individual accessions estimated in this study should facilitate further research and indicate that the analysis of a single plant or SSD line may have limited effectiveness due to the large number of genotypes present in a single accession. The general evaluation of accessions, as commonly used by genebanks, may also be of limited effectiveness, as a heterogeneous accession with poor overall tolerance to biotic or abiotic stresses may contain individuals with an exceptional combination of genetic variants, but their unique value is masked by the presence of other genotypes [126]. The results obtained here should be compared with earlier studies [55,64] in order to determine to what extent Moroccan landraces constitute a separate gene pool from Polish cultivars and landraces. This will also allow us to assess the success of the crossbreeding programs with the Moroccan gene pool carried out in the 1960s/70s and whether any part of this gene pool has survived in breeding material to the present day. Considering the increasing risk of drought in Poland every year, any work aimed at identifying materials carrying genetic variants associated with drought tolerance is of great importance. There is a high probability that this gene pool is distinct from the Polish one, and that the variability contained in the Moroccan landraces has not yet been used in Polish breeding programs, in which drought tolerance has not been a priority direction.

## 4. Materials and Methods

### 4.1. Plant Material

**Figure 12:**
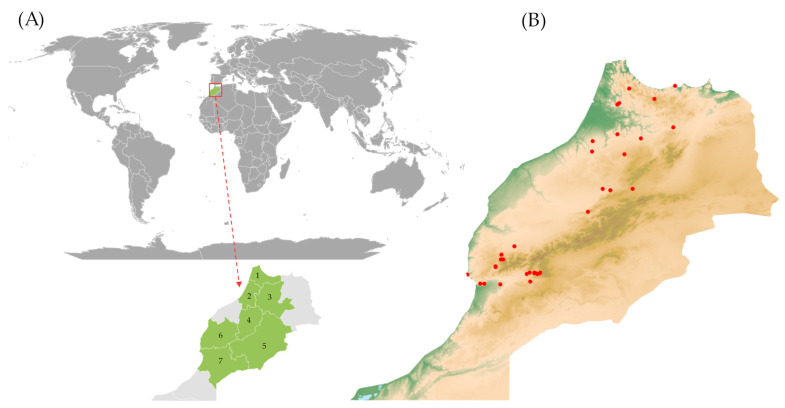
Maps. (**A**) The regions, where the expedition was carried out and from which the accessions surveyed had originated, were marked in maroon and numbered 1–7: 1—Tanger-Tétouan-Al Hoceïma; 2—Rabat-Salé-Kénitra; 3—Fès-Meknès; 4—Béni Mellal-Khénifra; 5—Drâa-Tafilalet; 6—Marrakech-Safi; 7—Souss-Massa, (**B**) relief map of Morocco with indication of barley landraces collection sites.

The analysis included 63 accessions from Morocco stored in the collection of the National Center for Plant Gene Resources (NCPGR), i.e., the Polish Genebank (Table S8). Forty eight accessions had landrace status and came from a field expedition conducted by the NCPGR team in 1986 and 1989 (Figure 12). Fifteen accessions with the status of breeding or research material were procured from other national and international institutes viz. Plant Breeding Station Strzelce (POL054), Plant Breeding Station Bąków (POL020), and Leibniz Institute of Plant Genetics and Crop Plant Research (DEU146). Originally, seven accessions came from the National Small Grains Germplasm Research Facility (USA029).

### 4.2. DNA Isolation

A leaf was taken from four-day-old, etiolated seedlings. Each accession was represented by eight randomly selected seedlings. DNA was isolated from the pooled tissue using a modified CTAB assay [127,128]. Qualitative and quantitative parameters were assessed using a NanoDrop ND-1000 spectrophotometer (NanoDrop Technologies, Willmington, DA, USA) and electrophoresis in a 1.5% agarose gel.

### 4.3. DArTseq Genotyping

Analysis of the genome representation using the next-generation sequencing (NGS)-based DArTseq technique was outsourced to the commercial laboratory Diversity Arrays Technology Pty Ltd. (http://www. diversityarrays.com, accessed on 1 September 2022). The resulting reads were mapped to the Morex barley genome assembly [50]. The matrix after SNPcalling is available on the Center for Open Science platform at https://osf.io/725nb/ (accessed on 10 October 2023).

### 4.4. Grain Morphometry

About 400 grains per accession were spilled on a Canon CanoScan LiDE 700 F desktop flatbed scanner for imaging at 300 dpi and saved as JPEG files. The files were subsequently analyzed using CSIRO GrainScan software [129]. This software uses automatic image recognition to identify an individual grain, providing the measurement in the graphic file for each grain. The following grain parameters were determined: area (mm^2^), perimeter (mm), length (mm), width (mm), and color (described in the device-independent 3D CIELAB color space as 3 RGB channels).

### 4.5. Eco-Geographical Data

For accessions with a known place of origin, data on climatic conditions at the collection site were downloaded. Data collected in the TerraClimate [130] database were used, which were downloaded using the Climate Toolbox developed by the Applied Climate Science Lab at the University of California, Merced (https://climatetoolbox.org/ accessed on 4 February 2023). The following data were downloaded: maximum temperature (tmax, average for month, units = °C), minimum temperature (tmin, average for month, units = °C), actual evapotranspiration (aet, monthly total, units = mm), climate water deficit (def, monthly total, units = mm), grass reference evapotranspiration (pet, monthly total, units = mm), precipitation (ppt, monthly total, units = mm), Palmer drought severity index (PDSI, at end of month, units = unitless), downward solar radiation flux at the surface (srad, monthly total, units = W/m^2^), water runoff (q, monthly total, units = mm), soil moisture (soil, monthly total, units = mm), and wind speed (ws, average for month, units = m/s).

The geographical distance was calculated using the geographical distance matrix generator v 1.2.3 [131] software.

### 4.6. Data Analysis

The variability of environmental parameters was assessed using raw meteorological data from 1958 to 2022. The significance of differences in climatic conditions at the collection sites was evaluated using the analysis of variance (ANOVA). The strength of the relationship between parameters was assessed using the Pearson correlation coefficient. A dissimilarity matrix was calculated using the Gower coefficient. Principal Component Analysis (PCA) was then performed. The statistical tests were performed using XLSTAT Ecology (Addinsoft, Inc., Brooklyn, NY, USA). Subsequently, multi-year averages for individual months and annual averages or totals were calculated for selected parameters.

Data of seven grain morphometric parameters were averaged across accessions. The minimum, maximum, mean, and standard deviation were then determined for each parameter within the study set. Averages within groups were compared using ANOVA and Tukey’s HSD post hoc test. The averaged data were normalized using unbiased standard deviation. As abovementioned, the dissimilarity matrix was calculated using the Gower coefficient and PCA was then performed. The statistical tests were performed using XLSTAT Ecology (Addinsoft, Inc., Brooklyn, NY, USA).

The results, in the form of a table listing SNPs that were detected in the accessions analyzed, were transformed to a binary matrix with their codominant character preserved as described by Dziurdziak et al. [64]. The results were then filtered for reproducibility (RepAvg ≥ 0.95), call rate (CallRate ≥ 0.95), and the minor allele frequency (MAF > 0. 01). For each locus, the PIC value was calculated. On the other hand, for each accession, the proportion of heterozygous loci was calculated (Ho), which, in the case of pooled samples, provides an estimation of accession heterogeneity, the unbiased expected heterozygosity (uHe) that measures the average gene diversity, and the fixation index (F), i.e., the inbreeding coefficient. Genetic distance was calculated based on the Jaccard coefficient. Principal Coordinate Analysis (PCoA) was performed. For groups separated based on biological status or eco-geographical data of the collection site, diversity parameters were evaluated, and the significance of differences was assessed using ANOVA and Tukey’s HSD *post hoc* test. As above, statistical tests were performed using XLSTAT Ecology (Addinsoft, Inc., Brooklyn, NY, USA). Population structure was assessed using an analysis of molecular variance (AMOVA) [51] and Bayesian model-based clustering implemented in STRUCTURE v 2. 3. 4 [132] with 5 × 10^4^ burn-ins and 15 × 10^4^ MCMCs in each run for five replications of K ranging from 1 to 15. To improve Bayesian clustering, the data were additionally filtered to meet the following criteria: RepAvg = 1.0, CallRate = 1.0, and MAF > 0.01. The number of clusters was determined based on the *posteriori* data probability for a given K and ΔK [52] and the full search algorithm implemented in CLUMPAK [53] was used to find the best match for replicated cluster analysis results. A cutoff value of 0.8 was set as the probability of assigning accession to the group.

Using passport data matrices of geographical distance (in km) and absolute elevation difference between intake sites were developed. The Mantel test with 10^5^ permutations was used to verify the correlation between genetic distance and all other dissimilarity matrices. A consensus configuration of genetic and grain morphometry data for accessions was obtained using Generalized Procrustes Analysis (GPA) [133] implemented in XLSTAT Ecology (Addinsoft, Inc., Brooklyn, NY, USA).

## 5. Conclusions

A characteristic feature of Moroccan landraces preserved in the Polish Genebank is high internal heterogeneity. Thus, a single accession contains a significant number of differentiated genotypes. This positive feature from the point of view of yield under unfavorable conditions, which is also beneficial for the conservation of biodiversity, is a major obstacle to the use of this germplasm in breeding programs. In order to make this unique material suitable for breeding, further research and selection of individuals with the most favorable combination of traits, supported by the most up-to-date technologies, will be necessary. The random selection of pure lines, as well as the general evaluation of accessions, will be affected by significant errors and may not provide satisfactory results, which will be reflected in the underutilization of this gene pool in Polish breeding. Considering the acute problem of drought in Poland and its impact on cereal yields, it is essential to support breeding with selected genotypes that have increased tolerance to water shortage. Moroccan landraces preserved in the Polish Genebank can be a valuable source of drought-adapted genotypes. The results presented here provide a roadmap for further research and implementation.

## Figures and Tables

**Table 1:** Summary of eco-geographical parameters in barley landraces for the entire year and growing season.

	Annual				Growing Season			
	Min.	Accession	Max.	Accession	Min.	Accession	Max.	Accession
tmax *	18.9	PL 42772	26.7	PL 42748	16	PL 42772	24.6	PL 42745
tmin *	4.7	PL 43341	14.4	PL 42745	1.7	PL 43341	12.1	PL 43340
ppt **	213.7	PL 42741	960.7	PL 43352	197.2	PL 42741	880.9	PL 43352
def **	759.1	PL 43352	1461.7	PL 42748	223.5	PL 43352	756.2	PL 42745
PDSI *	−0.89	PL 42766	−0.54	PL 43341	−0.84	PL 43348	−0.51	PL 43351
srad **	2450	PL 42766	2688.9	PL 42759	1464.5	PL 42766	1697.5	PL 42759
pet **	1285	PL 43350	1777.4	PL 42759	686.3	PL 43351	1002.1	PL 42759
q **	15.3	PL 42761	379.9	PL 43352	11.9	PL 42761	375.9	PL 43352
soil **	24.5	PL 42741	1017	PL 43352	20.2	PL 42741	883.8	PL 43352
ws *	2.8	PL 43349	4.6	PL 43340	2.7	PL 43348	4.5	PL 43340
aet **	191.7	PL 42741	586.9	PL 42766	175.7	PL 42741	495.97	PL 42766

* average, ** total, tmax—maximum temperature, tmin—minimum temperature, aet—actual evapotranspiration, def—climate water deficit, pet—grass reference evapotranspiration, ppt—precipitation, PDSI—Palmer drought severity index, srad—downward solar radiation flux at the surface, q—water runoff, soil—soil moisture, ws—wind speed.

**Table 2:** Summary of seven grain morphometric parameters. The accession number is in parentheses.

Parameter	Unit	Mean	Min.	Max.	Total var.	Breeding Material	Landrace
Surface area	mm^2^	24.90	20.62 (PL 42380)	28.54 (PL 42759)	8%	9%	6%
Perimeter	mm	31.62	25.38 (PL 42735)	34.63 (PL 42740)	6%	6%	5%
Length	mm	10.15	8.33 (PL 42735)	11.46 (PL 42740)	7%	6%	5%
Width	mm	3.10	2.92 (PL 42765)	3.36 (PL 42735)	3%	4%	3%
Color (Red)	8-bit/channel	164.54	156.11 (PL 42380)	185.83 (PL 40414)	3%	5%	2%
Color (Green)	8-bit/channel	138.80	130.09 (PL 42380)	161.83 (PL 40414)	3%	6%	2%
Color (Blue)	8-bit/channel	112.52	106.12 (PL 42380)	133.29 (PL 40414)	3%	6%	2%

The number in parentheses is according to Table S8.

**Table 3:** Summary of point mutation abundance at the loci tested by chromosome based on DArTseq SNP analysis of 63 Moroccan barley accessions.

			Abundance on Chromosomes							
			1H	2H	3H	4H	5H	6H	7H	Total
**Transitions**	**Purines**	**A > G**	192	259	232	178	275	189	233	1558
		**G > A**	195	248	220	147	235	180	219	1444
	**Pyrimidines**	**C > T**	181	233	249	161	252	175	237	1488
		**T > C**	140	230	238	145	224	135	213	1325
**Transversion**	**Purines > Pyrimidines**	**A > C**	35	68	65	48	70	48	74	408
		**A > T**	32	46	34	34	44	34	34	258
		**G > C**	103	122	122	86	122	89	110	754
		**G > T**	57	94	85	46	87	55	68	492
	**Pyrimidines > Purines**	**C > A**	49	86	76	47	61	52	77	448
		**C > G**	89	163	149	82	121	107	147	858
		**T > A**	25	42	42	25	49	27	38	248
		**T > G**	55	95	73	45	64	63	61	456
**% Ts**			61.41%	57.53%	59.24%	60.44%	61.47%	58.84%	59.70%	59.72%
**% Tv**			38.59%	42.47%	40.76%	39.56%	38.53%	41.16%	40.30%	40.28%
**Ts/Tv ratio**			1.59	1.35	1.45	1.53	1.60	1.43	1.48	1.48

**Table 4:** Summary of genes located around areas of unique SNP occurrence at 5H and 6H in accessions collected at sites with extreme Palmer drought severity index (PDSI) values.

chr	Region	Gene ID	uniProt	Protein
5H	370151444–371703455	HORVU5Hr1G047630	A0A287R3K4	Trichome birefringence-like N-terminal domain-containing protein
	552908671–552945241	HORVU5Hr1G077120	A8R7E1	Putative esterase/lipase/thioesterase family protein
		HORVU5Hr1G077150	M0Z0R4	Atos-like conserved domain-containing protein
6H	19490420–19611998	HORVU6Hr1G011030	Q9XGV5	Dehydrin 12
	170362846–172463480	HORVU6Hr1G035420	O23981	Cinnamyl alcohol dehydrogenase
	341714489–352940716	HORVU6Hr1G054420	Q9FEX6	coproporphyrinogen oxidase
	561295698–564061223	HORVU6Hr1G087200	J7QBS2	Putative proton-dependent oligopeptide/low-affinity nitrate transporter
		HORVU6Hr1G086940	A0A287V1V4	BFN domain-containing protein
		HORVU6Hr1G087190	F2E328	Histone H2B
		HORVU6Hr1G087090	F2E586	monogalactosyldiacylglycerol synthase
		HORVU6Hr1G086910	M0Y667	Serine-rich protein

## Data Availability

The raw data of DArTseq SNP used in this study are openly available on the platform Center for Open Science at https://osf.io/725nb/ (accessed on 10 October 2023) DOI 10.17605/OSF.IO/725NB.

## Supplementary Materials

The following supporting information can be downloaded at: https://www.mdpi.com/article/10.3390/ijms242216350/s1.
