# Supplementary material for: Characterization of the Moroccan Barley Germplasm Preserved in the Polish Genebank as a First Step towards Selecting Forms with Increased Drought Tolerance
Source: Int J Mol Sci. 2023 Nov 15;24(22):16350. doi: 10.3390/ijms242216350 (PMC10671370; doi:10.3390/ijms242216350)
Supplement: Supplementary file 1 [file ijms-24-16350-s001.zip › Supplement/Figures S.pdf]

**Figure S1.** Rotable 3D presentation of the Principal Component Analysis results for eco-geographic parameters in the places of origin of the 47 Moroccan barley landraces. Results in the first three components' system. Each point denotes one tested accession. Accession numbering according to Table 5. File: Figure S1.html

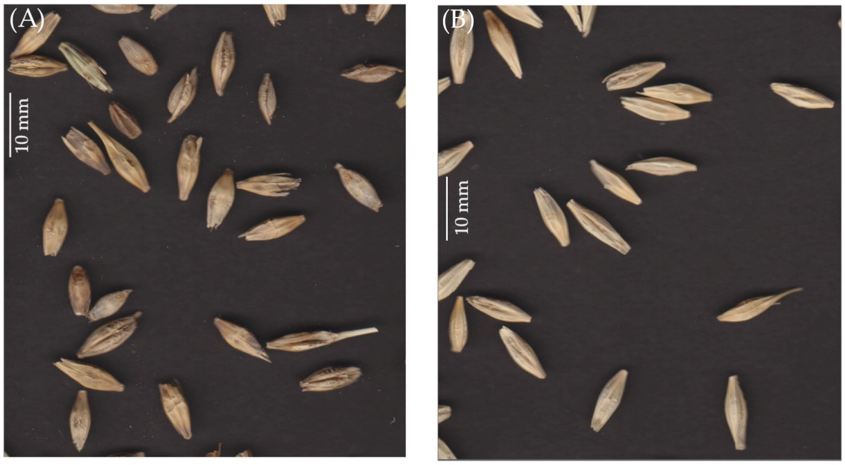


**Figure S2**. Grain color variation within Morrocan barley accessions: (**a**) PL 42380; (**b**) PL 40414.

**Figure S3.** Rotable 3D presentation of the Principal Component Analysis results for morphometric parameters of the 63 Moroccan barley accessions. Results in the first three components' system. Each point denotes one tested accession. Accessions numbering according to Table 5. File: Figure S3.html

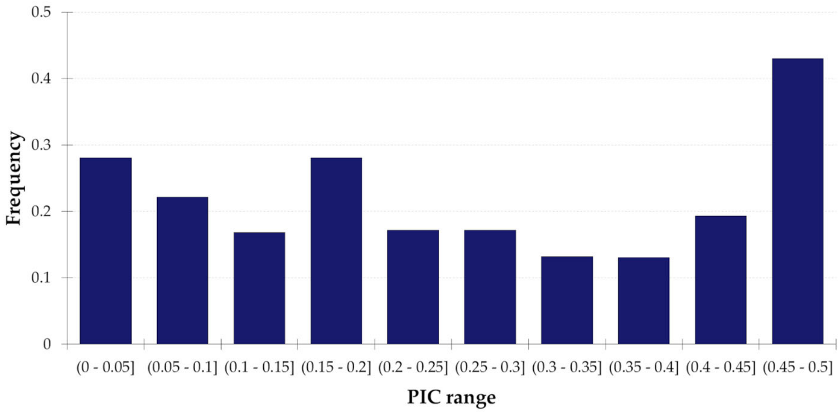


**Figure S4.** Summary of polymorphism information content (PIC) values. Range of relative frequencies for all analyzed DArTseq loci in 63 Morrocan barley accessions.

**Figure S5.** Rotable 3D presentation of the Principal Coordinate Analysis results for DArTseq data of 63 barley Morrocan accessions with indication their biological status. Results in the first three coordinates' system. Each point denotes one tested accession. Numbering according to Table 5. File: Figure S5.html

**Figure S6**. Rotable 3D presentation of the Principal Coordinate Analysis results for DArTseq data of 47 Morrocan barley landraces with indication their region of origin. Results in the first three coordinates' system. Each point denotes one tested accession. Numbering according to Table 5. File: Figure 6.html

Figure S7. Rotable 3D presentation of the Generalized Procrustes Analysis of genotypic and grain morphologic data of 63 Morrocan barley accessions. Results in the first three coordinates' system. Each point denotes one tested cultivar. Numbering according to Table 5. File: Figure S7.html
