# Supplementary material for: Characterization of the Moroccan Barley Germplasm Preserved in the Polish Genebank as a First Step towards Selecting Forms with Increased Drought Tolerance
Source: Int J Mol Sci. 2023 Nov 15;24(22):16350. doi: 10.3390/ijms242216350 (PMC10671370; doi:10.3390/ijms242216350)
Supplement: Supplementary file 1 [file ijms-24-16350-s001.zip › Supplement/Table S1-S5 and S7.pdf]

Table 1. Results of ANOVA analysis for eco-geographical data of the origin sites of barley landraces from Morocco

			Annual				Growing season			
	**Source**	**DF**	**Sum of squares**	**Mean squares**	**F**	**Pr > F**	**Sum of squares**	**Mean squares**	**F**	**Pr > F**
	Model	46	1993.24	43.33	1.03	0.427	1963.69	42.69	1.01	0.455
	Error	516	21772.50	42.19			21778.91	42.21		
**tmax**	Corrected Total	562	23765.74				23742.60			
	Model	46	3778.22	82.14	2.81	**< 0.0001**	3744.01	81.39	2.78	**< 0.0001**
	Error	516	15105.71	29.27			15102.43	29.27		
**tmin**	Corrected Total	562	18883.93				18846.44			
	Model	46	103818.21	2256.92	2.85	**< 0.0001**	103728.36	2254.96	2.84	**< 0.0001**
	Error	516	408585.25	791.83			409690.66	793.97		
**ppt**	Corrected Total	562	512403.46				513419.02			
	Model	46	136703.03	2971.80	0.58	0.989	136307.09	2963.20	0.58	0.989
	Error	516	2655331.94	5145.99			2658576.84	5152.28		
**def**	Corrected Total	562	2792034.96				2794883.93			
	Model	46	4.12	0.09	3.28	**< 0.0001**	4.14	0.09	3.29	**< 0.0001**
	Error	516	14.11	0.03			14.12	0.03		
**pdsi**	Corrected Total	562	18.23				18.26			
	Model	46	21000.18	456.53	0.11	1.000	22392.52	486.79	0.12	1.000
	Error	516	2092940.12	4056.09			2089550.35	4049.52		
**srad**	Corrected Total	562	2113940.30				2111942.87			
	Model	46	68741.81	1494.39	0.47	0.999	68938.89	1498.67	0.47	0.999
	Error	516	1640196.08	3178.67			1640006.40	3178.31		
**pet**	Corrected Total	562	1708937.88				1708945.29			
	Model	46	22677.62	492.99	3.97	**< 0.0001**	22668.29	492.79	3.97	**< 0.0001**
	Error	516	64045.25	124.12			64087.65	124.20		
**q**	Corrected Total	562	86722.87				86755.93			
	Model	46	182725.14	3972.29	27.31	**< 0.0001**	182798.60	3973.88	27.32	**< 0.0001**
	Error	516	75054.65	145.45			75051.98	145.45		
**soil**	Corrected Total	562	257779.79				257850.58			
	Model	46	95.81	2.08	10.14	**< 0.0001**	95.78	2.08	10.17	**< 0.0001**
	Error	516	105.95	0.21			105.67	0.20		
**ws**	Corrected Total	562	201.76				201.45			
	Model	46	34782.31	756.14	2.05	**0.000**	34671.34	753.72	2.04	**0.000**
	Error	516	190296.89	368.79			191036.84	370.23		
**aet**	Corrected Total	562	225079.21				225708.18			

Computed against model Y=Mean(Y), tmax - Maximum Temperature, tmin - Minimum Temperature, aet - Actual Evapotranspiration, def - Climate Water Deficit, pet - Grass Reference Evapotranspiration, ppt - Precipitation, PDSI - Palmer Drought Severity Index, srad - Downward Solar Radiation Flux at the Surface, q - Water Runoff, soil – Soil Moisture, ws - Wind Speed,

Table S2. Homogeneous groups based on ANOVA and Tukey's HSD post-hoc test

Accession	tmin	ppt	pdsi	q	soil	ws	aet
PL 40414	ABC	CD	ABC	DE	DE	CDE	ABCDE
PL 40672	ABC	CD	ABC	DE	DE	CDE	ABCDE
PL 40777	ABC	CD	ABC	DE	DE	CDE	ABCDE
PL 40979	BC	BCD	ABC	CDE	DE	BCDE	ABCDE
PL 40980	BC	BCD	ABC	CDE	DE	BCDE	ABCDE
PL 40981	A	D	ABC	E	E	ABCDE	E
PL 40982	AB	D	ABC	E	E	ABC	E
PL 40983	ABC	CD	ABC	DE	DE	CDE	ABCDE
PL 42378	ABC	CD	ABC	DE	DE	AB	DE
PL 42379	A	D	ABC	E	E	DE	E
PL 42380	ABC	CD	ABC	E	DE	CDE	BCDE
PL 42593	ABC	CD	ABC	E	DE	DE	DE
PL 42594	BC	BCD	ABC	CDE	DE	CDE	ABCDE
PL 42595	ABC	BCD	ABC	CDE	DE	CDE	ABCDE
PL 42694	ABC	BCD	ABC	CDE	DE	CDE	ABCDE
PL 42735	ABC	BCD	ABC	CDE	DE	CDE	ABCDE
PL 42736	ABC	BCD	ABC	CDE	DE	CDE	ABCDE
PL 42737	ABC	BCD	ABC	CDE	DE	CDE	ABCDE
PL 42738	ABC	BCD	ABC	CDE	DE	CDE	ABCDE
PL 42739	ABC	BCD	ABC	CDE	DE	CDE	ABCDE
PL 42740	ABC	BCD	ABC	CDE	DE	CDE	ABCDE
PL 42741	ABC	CD	ABC	DE	DE	BCDE	ABCDE
PL 42742	BC	BCD	ABC	CDE	DE	CDE	ABCDE
PL 42743	BC	CD	ABC	E	DE	CDE	DE
PL 42744	ABC	BCD	ABC	CDE	D	E	ABCDE
PL 42745	ABC	BCD	ABC	CDE	DE	CDE	ABCDE
PL 42747	ABC	BCD	ABC	BCDE	DE	E	ABCDE
PL 42748	ABC	BCD	ABC	BCDE	DE	DE	ABCDE
PL 42749	ABC	AB	C	ABCD	AB	E	A
PL 42750	ABC	AB	BC	ABC	B	DE	AB
PL 42751	BC	BCD	ABC	CDE	DE	ABCD	ABCDE
PL 42752	ABC	CD	ABC	DE	DE	AB	CDE
PL 42753	ABC	CD	ABC	DE	DE	AB	DE
PL 42754	ABC	BCD	ABC	CDE	DE	CDE	ABCDE
PL 42755	A	D	ABC	E	DE	A	E
PL 42756	C	BCD	ABC	CDE	DE	CDE	ABCDE
PL 42757	ABC	CD	ABC	E	DE	CDE	ABCDE
PL 42759	ABC	CD	ABC	E	DE	CDE	ABCDE
PL 42760	ABC	CD	ABC	E	DE	CDE	ABCDE
PL 42761	ABC	ABC	ABC	BCDE	DE	DE	ABCD
PL 42762	ABC	BCD	ABC	CDE	DE	DE	ABCDE
PL 42763	ABC	ABCD	ABC	BCDE	D	DE	ABCD
PL 42764	ABC	BCD	C	CDE	DE	E	ABCDE
PL 42765	ABC	ABCD	ABC	BCDE	DE	E	ABCDE
PL 42766	AB	CD	AB	E	DE	ABCD	ABCDE
PL 42767	ABC	AB	A	AB	C	ABC	ABCD
PL 42772	ABC	A	ABC	A	A	BCDE	ABC

tmin - Minimum Temperature, ppt - Precipitation, pdsi - Palmer Drought Severity Index, q - Water Runoff, soil – Soil Moisture, ws - Wind Speed.

Table S3. Results of the correlation analysis of eco-geographical parameters during the growing season in the origin sites of barley landraces in Morocco.

Variables	tmin	ppt	PDSI	q	soil	ws	aet	alt
tmin	1							
ppt	-0.079	1						
PDSI	**-0.395**	-0.106	1					
q	0.055	**0.956**	-0.044	1				
soil	0.147	**0.872**	-0.043	**0.889**	1			
ws	0.096	-0.285	0.228	-0.136	-0.117	1		
aet	-0.219	**0.953**	-0.157	**0.823**	**0.762**	**-0.414**	1	
alt	**-0.811**	0.055	**0.329**	-0.055	-0.208	-0.217	0.177	1

Values in bold are different from 0 with a significance level alpha=0.05, tmin - Minimum Temperature, aet - Actual Evapotranspiration, ppt - Precipitation, PDSI - Palmer Drought Severity Index, q - Water Runoff, soil - Soil Moisture, ws - Wind Speed, alt – Altitude of a collection site.

Table S4. Correlation analysis results of grain morphometric parameters for 64 Moroccan accessions.

Variables	area	perimeter	length	width	grain ch1	grain ch2	grain ch3
area	1						
perimeter	**0.907**	1					
length	**0.921**	**0.966**	1				
width	**0.363**	0.009	-0.013	1			
grain ch1	**0.251**	-0.009	0.154	**0.376**	1		
grain ch2	0.219	-0.008	0.147	**0.315**	**0.977**	1	
grain ch3	0.000	-0.155	-0.036	0.182	**0.833**	**0.911**	1

Values in bold are different from 0 with a significance level alpha=0.05, tmin - Minimum Temperature, aet - Actual Evapotranspiration, ppt - Precipitation, PDSI - Palmer Drought Severity Index, q - Water Runoff, soil - Soil Moisture, ws - Wind Speed, alt – Altitude of a collection site.

Table 1. Summary of DArTseq loci distribution on chromosomes for 63 Morrocan barley acecessions. Chromosome lengths according to barley genome assembly: IBSC_v2 [50].

	no. Loci	Length [Mbp]	Mean distance [Mbp]	mean PIC
1H	1153	558.54	0.48	0.25
2H	1686	768.08	0.46	0.26
3H	1585	699.71	0.44	0.28
4H	1044	647.06	0.62	0.27
5H	1604	670.03	0.42	0.25
6H	1154	583.38	0.51	0.26
7H	1511	657.22	0.43	0.25
Total	9737	4584.02	0.47	0.26

Table S6. Separate xlsx file.

Table S7. Summary of unique allels by chromosome based on DArTseq analysis of 63 Morrocan barley accession and under extreme and significantly different environmental conditions at the collection sites.

	Abundance on chromosomes							
**Variables**	1H	2H	3H	4H	5H	6H	7H	Total
**tmin**	12	14	11	4	12	14	9	76
**ppt**	20	20	23	17	19	25	23	147
**PDSI**	10	18	16	16	16	38	15	129
**q**	0	4	1	1	0	2	4	12
**soil**	20	26	23	19	20	30	26	164
**ws**	0	7	1	1	2	2	2	15
**aet**	5	5	9	4	11	3	5	42
**alt**	0	1	1	0	4	1	0	7

tmin - Minimum Temperature, ppt - Precipitation, PDSI - Palmer Drought Severity Index, q - Water Runoff, soil - Soil Moisture, ws - Wind Speed, alt – Altitude of a collection site.
