# Supplementary material for: Characterization of the Moroccan Barley Germplasm Preserved in the Polish Genebank as a First Step towards Selecting Forms with Increased Drought Tolerance
Source: Int J Mol Sci. 2023 Nov 15;24(22):16350. doi: 10.3390/ijms242216350 (PMC10671370; doi:10.3390/ijms242216350)
Supplement: Supplementary file 1 [file ijms-24-16350-s001.zip › Supplement/Tables S.pdf]

Table 1. Results of ANOVA analysis for eco-geographical data of the origin sites of barley landraces from Morocco

			Annual				Growing season			
	**Source**	**DF**	**Sum of squares**	**Mean squares**	**F**	**Pr > F**	**Sum of squares**	**Mean squares**	**F**	**Pr > F**
	Model	46	1993.24	43.33	1.03	0.427	1963.69	42.69	1.01	0.455
	Error	516	21772.50	42.19			21778.91	42.21		
**tmax**	Corrected Total	562	23765.74				23742.60			
	Model	46	3778.22	82.14	2.81	**< 0.0001**	3744.01	81.39	2.78	**< 0.0001**
	Error	516	15105.71	29.27			15102.43	29.27		
**tmin**	Corrected Total	562	18883.93				18846.44			
	Model	46	103818.21	2256.92	2.85	**< 0.0001**	103728.36	2254.96	2.84	**< 0.0001**
	Error	516	408585.25	791.83			409690.66	793.97		
**ppt**	Corrected Total	562	512403.46				513419.02			
	Model	46	136703.03	2971.80	0.58	0.989	136307.09	2963.20	0.58	0.989
	Error	516	2655331.94	5145.99			2658576.84	5152.28		
**def**	Corrected Total	562	2792034.96				2794883.93			
	Model	46	4.12	0.09	3.28	**< 0.0001**	4.14	0.09	3.29	**< 0.0001**
	Error	516	14.11	0.03			14.12	0.03		
**pdsi**	Corrected Total	562	18.23				18.26			
	Model	46	21000.18	456.53	0.11	1.000	22392.52	486.79	0.12	1.000
	Error	516	2092940.12	4056.09			2089550.35	4049.52		
**srad**	Corrected Total	562	2113940.30				2111942.87			
	Model	46	68741.81	1494.39	0.47	0.999	68938.89	1498.67	0.47	0.999
	Error	516	1640196.08	3178.67			1640006.40	3178.31		
**pet**	Corrected Total	562	1708937.88				1708945.29			
	Model	46	22677.62	492.99	3.97	**< 0.0001**	22668.29	492.79	3.97	**< 0.0001**
	Error	516	64045.25	124.12			64087.65	124.20		
**q**	Corrected Total	562	86722.87				86755.93			
	Model	46	182725.14	3972.29	27.31	**< 0.0001**	182798.60	3973.88	27.32	**< 0.0001**
	Error	516	75054.65	145.45			75051.98	145.45		
**soil**	Corrected Total	562	257779.79				257850.58			
	Model	46	95.81	2.08	10.14	**< 0.0001**	95.78	2.08	10.17	**< 0.0001**
	Error	516	105.95	0.21			105.67	0.20		
**ws**	Corrected Total	562	201.76				201.45			
	Model	46	34782.31	756.14	2.05	**0.000**	34671.34	753.72	2.04	**0.000**
	Error	516	190296.89	368.79			191036.84	370.23		
**aet**	Corrected Total	562	225079.21				225708.18			

Computed against model Y=Mean(Y), tmax - Maximum Temperature, tmin - Minimum Temperature, aet - Actual Evapotranspiration, def - Climate Water Deficit, pet - Grass Reference Evapotranspiration, ppt - Precipitation, PDSI - Palmer Drought Severity Index, srad - Downward Solar Radiation Flux at the Surface, q - Water Runoff, soil – Soil Moisture, ws - Wind Speed,

Table S2. Homogeneous groups based on ANOVA and Tukey's HSD post-hoc test

Accession	tmin	ppt	pdsi	q	soil	ws	aet
PL 40414	ABC	CD	ABC	DE	DE	CDE	ABCDE
PL 40672	ABC	CD	ABC	DE	DE	CDE	ABCDE
PL 40777	ABC	CD	ABC	DE	DE	CDE	ABCDE
PL 40979	BC	BCD	ABC	CDE	DE	BCDE	ABCDE
PL 40980	BC	BCD	ABC	CDE	DE	BCDE	ABCDE
PL 40981	A	D	ABC	E	E	ABCDE	E
PL 40982	AB	D	ABC	E	E	ABC	E
PL 40983	ABC	CD	ABC	DE	DE	CDE	ABCDE
PL 42378	ABC	CD	ABC	DE	DE	AB	DE
PL 42379	A	D	ABC	E	E	DE	E
PL 42380	ABC	CD	ABC	E	DE	CDE	BCDE
PL 42593	ABC	CD	ABC	E	DE	DE	DE
PL 42594	BC	BCD	ABC	CDE	DE	CDE	ABCDE
PL 42595	ABC	BCD	ABC	CDE	DE	CDE	ABCDE
PL 42694	ABC	BCD	ABC	CDE	DE	CDE	ABCDE
PL 42735	ABC	BCD	ABC	CDE	DE	CDE	ABCDE
PL 42736	ABC	BCD	ABC	CDE	DE	CDE	ABCDE
PL 42737	ABC	BCD	ABC	CDE	DE	CDE	ABCDE
PL 42738	ABC	BCD	ABC	CDE	DE	CDE	ABCDE
PL 42739	ABC	BCD	ABC	CDE	DE	CDE	ABCDE
PL 42740	ABC	BCD	ABC	CDE	DE	CDE	ABCDE
PL 42741	ABC	CD	ABC	DE	DE	BCDE	ABCDE
PL 42742	BC	BCD	ABC	CDE	DE	CDE	ABCDE
PL 42743	BC	CD	ABC	E	DE	CDE	DE
PL 42744	ABC	BCD	ABC	CDE	D	E	ABCDE
PL 42745	ABC	BCD	ABC	CDE	DE	CDE	ABCDE
PL 42747	ABC	BCD	ABC	BCDE	DE	E	ABCDE
PL 42748	ABC	BCD	ABC	BCDE	DE	DE	ABCDE
PL 42749	ABC	AB	C	ABCD	AB	E	A
PL 42750	ABC	AB	BC	ABC	B	DE	AB
PL 42751	BC	BCD	ABC	CDE	DE	ABCD	ABCDE
PL 42752	ABC	CD	ABC	DE	DE	AB	CDE
PL 42753	ABC	CD	ABC	DE	DE	AB	DE
PL 42754	ABC	BCD	ABC	CDE	DE	CDE	ABCDE
PL 42755	A	D	ABC	E	DE	A	E
PL 42756	C	BCD	ABC	CDE	DE	CDE	ABCDE
PL 42757	ABC	CD	ABC	E	DE	CDE	ABCDE
PL 42759	ABC	CD	ABC	E	DE	CDE	ABCDE
PL 42760	ABC	CD	ABC	E	DE	CDE	ABCDE
PL 42761	ABC	ABC	ABC	BCDE	DE	DE	ABCD
PL 42762	ABC	BCD	ABC	CDE	DE	DE	ABCDE
PL 42763	ABC	ABCD	ABC	BCDE	D	DE	ABCD
PL 42764	ABC	BCD	C	CDE	DE	E	ABCDE
PL 42765	ABC	ABCD	ABC	BCDE	DE	E	ABCDE
PL 42766	AB	CD	AB	E	DE	ABCD	ABCDE
PL 42767	ABC	AB	A	AB	C	ABC	ABCD
PL 42772	ABC	A	ABC	A	A	BCDE	ABC

tmin - Minimum Temperature, ppt - Precipitation, pdsi - Palmer Drought Severity Index, q - Water Runoff, soil – Soil Moisture, ws - Wind Speed.

Table S3. Results of the correlation analysis of eco-geographical parameters during the growing season in the origin sites of barley landraces in Morocco.

**Variables**	**tmin**	**ppt**	**PDSI**	**q**	**soil**	**ws**	**aet**	**alt**
tmin	1							
ppt	-0.079	1						
PDSI	**-0.395**	-0.106	1					
q	0.055	**0.956**	-0.044	1				
soil	0.147	**0.872**	-0.043	**0.889**	**1**			
ws	0.096	-0.285	0.228	-0.136	-0.117	1		
aet	-0.219	**0.953**	-0.157	**0.823**	**0.762**	**-0.414**	1	
alt	**-0.811**	0.055	**0.329**	-0.055	-0.208	-0.217	0.177	1

Values in bold are different from 0 with a significance level alpha=0.05, tmin - Minimum Temperature, aet - Actual Evapotranspiration, ppt - Precipitation, PDSI - Palmer Drought Severity Index, q - Water Runoff, soil - Soil Moisture, ws - Wind Speed, alt – Altitude of a collection site.

Table S4. Correlation analysis results of grain morphometric parameters for 64 Moroccan accessions.

Variables	area	perimeter	length	width	grain ch1	grain ch2	grain ch3
area	1						
perimeter	**0.907**	1					
length	**0.921**	**0.966**	1				
width	**0.363**	0.009	-0.013	**1**			
grain ch1	**0.251**	-0.009	0.154	**0.376**	**1**		
grain ch2	0.219	-0.008	0.147	**0.315**	**0.977**	**1**	
grain ch3	0.000	-0.155	-0.036	0.182	**0.833**	**0.911**	**1**

Values in bold are different from 0 with a significance level alpha=0.05, tmin - Minimum Temperature, aet - Actual Evapotranspiration, ppt - Precipitation, PDSI - Palmer Drought Severity Index, q - Water Runoff, soil - Soil Moisture, ws - Wind Speed, alt – Altitude of a collection site.

Table 1. Summary of DArTseq loci distribution on chromosomes for 63 Morrocan barley acecessions. Chromosome lengths according to barley genome assembly: IBSC_v2 [50].

	**no. Loci**	**Length [Mbp]**	**Mean distance [Mbp]**	**mean PIC**
1H	1153	558.54	0.48	0.25
2H	1686	768.08	0.46	0.26
3H	1585	699.71	0.44	0.28
4H	1044	647.06	0.62	0.27
5H	1604	670.03	0.42	0.25
6H	1154	583.38	0.51	0.26
7H	1511	657.22	0.43	0.25
Total	9737	4584.02	0.47	0.26

Table S6. Separate xlsx file.

Table S7. Summary of unique allels by chromosome based on DArTseq analysis of 63 Morrocan barley accession and under extreme and significantly different environmental conditions at the collection sites.

	Abundance on chromosomes							
**Variables**	1H	2H	3H	4H	5H	6H	7H	Total
**tmin**	12	14	11	4	12	14	9	76
**ppt**	20	20	23	17	19	25	23	147
**PDSI**	10	18	16	16	16	38	15	129
**q**	0	4	1	1	0	2	4	12
**soil**	20	26	23	19	20	30	26	164
**ws**	0	7	1	1	2	2	2	15
**aet**	5	5	9	4	11	3	5	42
**alt**	0	1	1	0	4	1	0	7

tmin - Minimum Temperature, ppt - Precipitation, PDSI - Palmer Drought Severity Index, q - Water Runoff, soil - Soil Moisture, ws - Wind Speed, alt – Altitude of a collection site.

Table S8. The list of Maroccan barley accessions from the collection of the National Center for Plant Gene Resources which were selected for DArTseq analysis.

	Accession number	Acquisition year	Latitude	Longitude	Altitude	Status	Donor Code
1	PL 40414	1972				research/breeding material	POL054
2	PL 40672	1973				research/breeding material	DEU146
3	PL 40777	1972				research/breeding material	POL020
4	PL 40979	1972				research/breeding material	POL020
5	PL 40980	1972				research/breeding material	POL020
6	PL 40981	1972				research/breeding material	POL020
7	PL 40982	1972				research/breeding material	POL020
8	PL 40983	1972				research/breeding material	DEU146
9	PL 42378	1984				research/breeding material	POL020
10	PL 42379	1984				research/breeding material	POL020
11	PL 42380	1984				research/breeding material	POL020
12	PL 42593	1984				research/breeding material	POL020
13	PL 42594	1984				research/breeding material	POL020
14	PL 42595	1984				research/breeding material	POL020
15	PL 42694	1984				research/breeding material	POL020
16	PL 42735	1986				landrace/traditional cultivar	
17	PL 42736	1986	31°22N	8°31W	550	landrace/traditional cultivar	
18	PL 42737	1986	31°22N	8°31W	550	landrace/traditional cultivar	
19	PL 42738	1986	31°10N	8°53W	550	landrace/traditional cultivar	
20	PL 42739	1986	31°03N	8°50W	1550	landrace/traditional cultivar	
21	PL 42740	1986	31°03N	8°50W	1550	landrace/traditional cultivar	
22	PL 42741	1986	30°28N	9°22W	150	landrace/traditional cultivar	
23	PL 42742	1986	30°28N	9°29W	350	landrace/traditional cultivar	
24	PL 42743	1986	31°10N	8°53W	550	landrace/traditional cultivar	
25	PL 42744	1986	30°52N	9°03W	250	landrace/traditional cultivar	
26	PL 42745	1986	30°27N	8°55W	250	landrace/traditional cultivar	
27	PL 42747	1986	30°44N	8°05W	1000	landrace/traditional cultivar	
28	PL 42748	1986	30°42N	8°10W	750	landrace/traditional cultivar	
29	PL 42749	1986	30°43N	7°48W	1650	landrace/traditional cultivar	
30	PL 42750	1986	30°42N	7°52W	1350	landrace/traditional cultivar	
31	PL 42751	1986	30°42N	7°52W	1350	landrace/traditional cultivar	
32	PL 42752	1986	30°42N	7°52W	1350	landrace/traditional cultivar	
33	PL 42753	1986	30°42N	7°52W	1350	landrace/traditional cultivar	
34	PL 42754	1986	30°42N	7°52W	1350	landrace/traditional cultivar	

35	PL 42755	1986	30°42N	7°52W	1350	landrace/traditional cultivar
36	PL 42756	1986	30°42N	7°52W	1350	landrace/traditional cultivar
37	PL 42757	1986	30°42N	7°52W	1350	landrace/traditional cultivar
38	PL 42759	1986	30°31N	8°04W	1350	landrace/traditional cultivar
39	PL 42760	1986	30°43N	7°48W	1650	landrace/traditional cultivar
40	PL 42761	1986	32°45N	5°10W	1450	landrace/traditional cultivar
41	PL 42762	1986	33°58N	4°56W	550	landrace/traditional cultivar
42	PL 42763	1986	33°35N	5°24W	550	landrace/traditional cultivar
43	PL 42764	1986	34°04N	5°36W	350	landrace/traditional cultivar
44	PL 42765	1986	33°54N	6°18W	200	landrace/traditional cultivar
45	PL 42766	1986	34°49N	5°33W	350	landrace/traditional cultivar
46	PL 42767	1986	34°47N	5°36W	450	landrace/traditional cultivar
47	PL 42772	1986	31°03N	8°54W	1350	landrace/traditional cultivar
48	PL 42969	1986	30°53N	9°03W	350	landrace/traditional cultivar
49	PL 42983	1986	30°52N	9°03W	250	landrace/traditional cultivar
50	PL 42984	1986	30°42N	7°52W	1350	landrace/traditional cultivar
51	PL 43340	1989	30°41N	9°51W	100	landrace/traditional cultivar
52	PL 43341	1989	30°44N	7°47W	1350	landrace/traditional cultivar
53	PL 43342	1989	30°44N	7°57W	1000	landrace/traditional cultivar
54	PL 43343	1992	30°43N	7°57W	1100	landrace/traditional cultivar
55	PL 43344	1989	30°43N	7°55W	1450	landrace/traditional cultivar
56	PL 43345	1989	32°27N	6°01W	1500	landrace/traditional cultivar
57	PL 43346	1989	32°12N	6°26W	700	landrace/traditional cultivar
58	PL 43347	1989	32°43N	5°48W	1000	landrace/traditional cultivar
59	PL 43348	1989	33°39N	6°19W	350	landrace/traditional cultivar
60	PL 43349	1989	34°14N	4°01W	1650	landrace/traditional cultivar
61	PL 43350	1989	35°14N	3°58W	200	landrace/traditional cultivar
62	PL 43351	1989	34°55N	4°33W	1200	landrace/traditional cultivar
63	PL 43352	1989	35°10N	5°16W	350	landrace/traditional cultivar
