# Supplementary figures and images for: Characterization of the Moroccan Barley Germplasm Preserved in the Polish Genebank as a First Step towards Selecting Forms with Increased Drought Tolerance
Source: Int J Mol Sci. 2023 Nov 15;24(22):16350. doi: 10.3390/ijms242216350 (PMC10671370; doi:10.3390/ijms242216350)

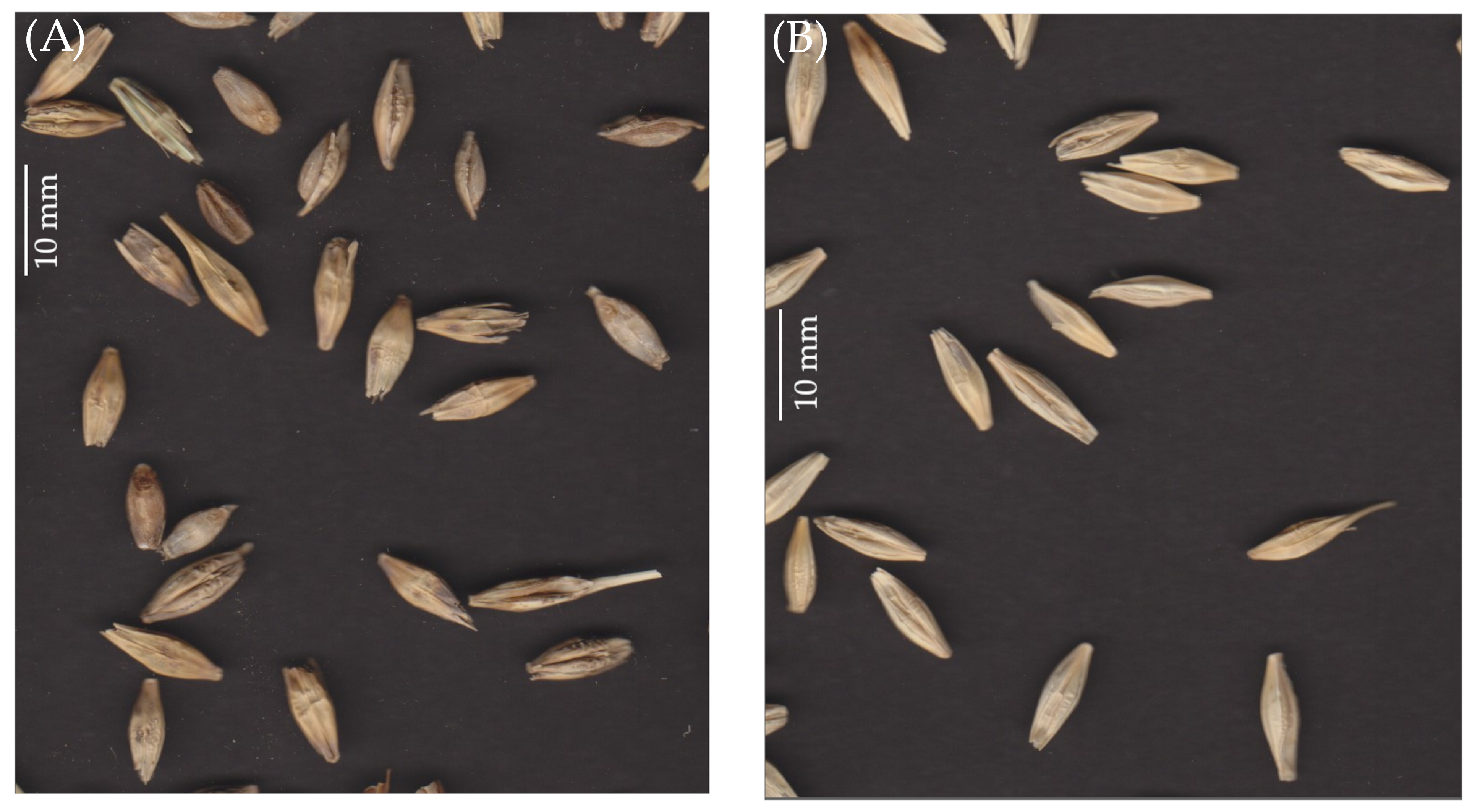

Supplement: Supplementary file 1 [file ijms-24-16350-s001.zip › Supplement/Figure S2.png]

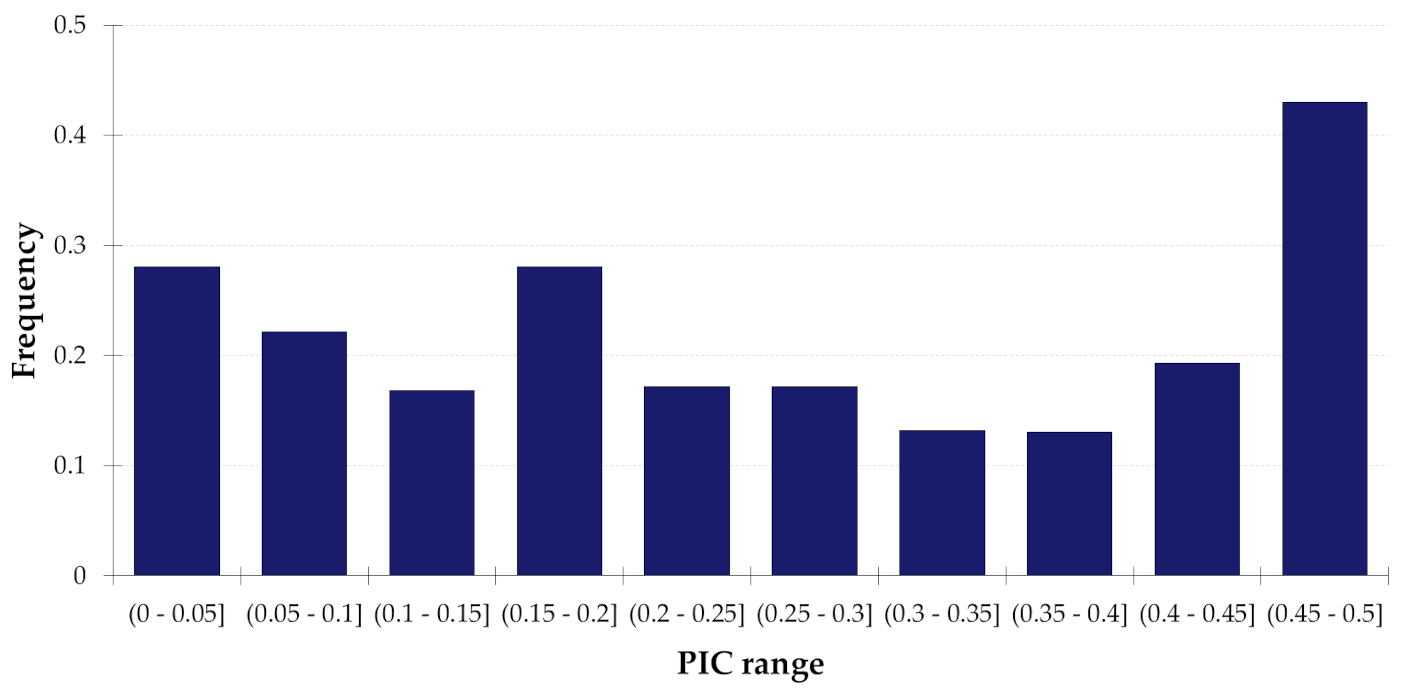

Supplement: Supplementary file 1 [file ijms-24-16350-s001.zip › Supplement/Figure S4.png]
